# A Series of Isatin-Hydrazones with Cytotoxic Activity and CDK2 Kinase Inhibitory Activity: A Potential Type II ATP Competitive Inhibitor

**DOI:** 10.3390/molecules25194400

**Published:** 2020-09-25

**Authors:** Huda S. Al-Salem, Md Arifuzzaman, Hamad M. Alkahtani, Ashraf N. Abdalla, Iman S. Issa, Aljawharah Alqathama, Fatemah S. Albalawi, A. F. M. Motiur Rahman

**Affiliations:** 1 Department of Pharmaceutical Chemistry, College of Pharmacy, King Saud University, Riyadh 11451, Saudi Arabia; ahamad@ksu.edu.sa (H.M.A.); iman_issa69@yahoo.com (I.S.I.); fsalbalawi@ksu.edu.sa (F.S.A.); 2 College of Pharmacy, Yeungnam University, Gyeongsan 38541, Korea; arifmilon2016@gmail.com; 3 Department of Pharmacology and Toxicology, Faculty of Pharmacy, Umm Al-Qura University, Makkah 21955, Saudi Arabia; ashraf_abdalla@hotmail.com; 4 Department of Pharmacognosy, Faculty of Pharmacy, Umm Al-Qura University, Makkah 21955, Saudi Arabia; aaqathama@uqu.edu.sa

**Keywords:** isatin-hydrazones, cytotoxicity, CDK2 inhibitor, ATP competitive inhibitor, ADME analysis

## Abstract

Isatin derivatives potentially act on various biological targets. In this article, a series of novel isatin-hydrazones were synthesized in excellent yields. Their cytotoxicity was tested against human breast adenocarcinoma (MCF7) and human ovary adenocarcinoma (A2780) cell lines using MTT assay. Compounds **4j** (IC_50_ = 1.51 ± 0.09 µM) and **4k** (IC_50_ = 3.56 ± 0.31) showed excellent activity against MCF7, whereas compound **4e** showed considerable cytotoxicity against both tested cell lines, MCF7 (IC_50_ = 5.46 ± 0.71 µM) and A2780 (IC_50_ = 18.96± 2.52 µM), respectively. Structure-activity relationships (SARs) revealed that, halogen substituents at 2,6-position of the C-ring of isatin-hydrazones are the most potent derivatives. In-silico absorption, distribution, metabolism and excretion (ADME) results demonstrated recommended drug likeness properties. Compounds **4j** (IC_50_ = 0.245 µM) and **4k** (IC_50_ = 0.300 µM) exhibited good inhibitory activity against the cell cycle regulator CDK2 protein kinase compared to imatinib (IC_50_ = 0.131 µM). A molecular docking study of **4j** and **4k** confirmed both compounds as type II ATP competitive inhibitors that made interactions with ATP binding pocket residues, as well as lacking interactions with active state DFG motif residues.

## 1. Introduction

Development of anticancer drugs is essential due to the increasing number of morbidity and mortality by cancer day-by-day all over the world. According to the International Agency for Research on Cancer, in 2018, around 18 million people were infected; 9.6 million people among them had died due to life threatening cancer [1,2]. It is rather alarming that cancer morbidity cases may increase to 29.5 million by 2040 [3]. Having said that, it is very much challenging to develop an anticancer drug due to the long and expensive synthesis/isolation process and the huge lack of opportunities to conduct clinical trials. Moreover, most of the anticancer drugs currently available are lacking specificity and have adverse effects. In this context, developing novel anticancer agents with great efficacy and high specificity becomes imperative. To overcome these challenges, researchers should develop a drug molecule with potent biological activity and low/no toxicity, study its mode of action, in silico properties and in vitro/vivo metabolism, conduct a toxicity evaluation [4,5], study its topoisomerase inhibitory activity [6,7,8] and enzyme inhibitory activity [9], etc., all of which are some of the key evaluation practices for the development of potential anticancer therapeutics. Regarding enzyme inhibitory activities, cyclin-dependent kinases (CDKs) are considered as a vital feature, inciting various key transitions in the cell cycle for cancer cells, in addition to instructing apoptosis, transcription and exocytosis. CDKs are active only when bound to their regulator proteins, cyclins. CDK activity is tightly controlled for successful cell division. Since abnormal cell division represents cancer pathology, controlling CDK activity has been shown as a promising therapeutic strategy. In particular, CDK2 plays an important role in DNA replication. Therefore, therapeutic strategies based on the inhibition of CDKs work as an encouraging viewpoint for anticancer drug discovery. With that being said, to consider a compound, such as a drug molecule, as a treatment, it is still necessary to first test their drug likeness properties as well as analyze their physiological descriptors, such as absorption, distribution, metabolism and excretion (ADME). ADME is an important physiological descriptor of chemical compounds used for selecting potential drug targets. However, testing a wide range of compounds directly in the clinical or pre-clinical phase is extensively time consuming and costly. Moreover, ADME is considered as the last step of drug development, where many drugs (approximately 60%) fail after all the procedures. To tackle these problems, recent experiments have utilized in silico ADME tools as the first step to shortlist the amount of target compounds by calculating predicted ADME properties and discarding the compounds with unsatisfactory ADME values from the drug designing pipeline [10]. 

**Figure 1:**
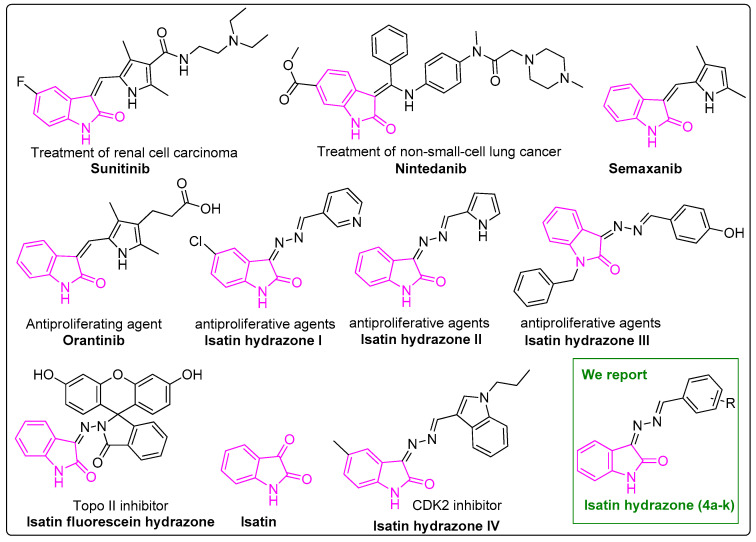
Isatin moiety containing active and potential drugs.

Isatin (**1**) is an organic compound first discovered in 1840 by Erdmann and Laurent from the oxidation of indigo dye [11,12]. It was considered as a synthetic product until isolated from natural sources, such as *Couroupita guianensis* [13], *Isatis tinctoria* [14] and *Calanthe discolor* [15], and from many other sources [16,17,18]. It has been reported that tryptophan obtained from food sources is usually converted to indole by gastrointestinal bacteria, which is further oxidized in the liver by CYP450 to isatin, therefore, isatin is present as an endogenous molecule in humans [19,20]. Various substituents on the isatin nucleus displayed numerous biological activities [21,22,23,24,25,26,27,28,29,30,31,32,33,34,35,36], including antimicrobial activity[31,37], topoisomerase inhibitory activity [7,38], epidermal growth factor receptor (EGFR) inhibitory activity [39], inhibitory activities on histone deacetylase (HDAC) [40,41], carbonic anhydrase [42,43,44], tyrosine kinase [45,46,47], cyclin-dependent kinases (CDKs) [9,48,49], adenylate cyclase inhibition [50] and protein tyrosine phosphatase (Shp2) [51]. A number of isatin-based marketed drugs and potential anticancer agents [41] are illustrated in Figure 1. Considering the importance of the development of anticancer therapeutics and the various biological properties of isatin and isatin nucleus-containing derivatives, a series of isatin-hydrazones were designed and synthesized, their cytotoxicities against two different cancer cell lines, namely MCF7 (human breast adenocarcinoma) and A2780 (human ovary adenocarcinoma), were evaluated, their structure–activity relationships (SARs) were studied, their ADME properties were studied using in silico ADME tools and cyclin-dependent kinases 2 inhibitory activities were performed using an enzyme inhibition assay. Additionally, docking simulations were conducted in order to explore the behavior of the synthesized compounds within the active site of CDK2 to justify its binding mechanism. 

## 2. Results and Discussion

### 2.1. Synthesis of Isatin-Hydrazones (***4***) 

**Scheme 1:**
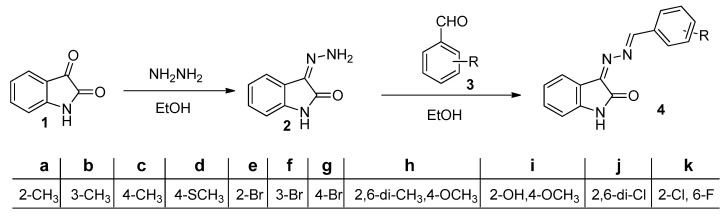
Synthesis of 3-((substituted)benzylidene)hydrazono)indolin-2-one (4).

Synthesis of 3-((substituted)benzylidene)hydrazono)indolin-2-one (**4**) was straightforward, as illustrated in Scheme 1 [36,52]. In the first step, a mixture of isatin (**1**) and hydrazine hydrate was refluxed in ethanol and isatin monohydrazone (**2**) was obtained in quantitative yields (~99%). Subsequently, the isatin monohydrazone (**2**) was refluxed with substituted aryl aldehydes (**3**) in the presence of a catalytic amount of glacial acetic acid in absolute ethanol to obtain 3-((substituted)benzylidene)hydrazono)indolin-2-one (**4**) in good to excellent yields (75–98%). The structures of the synthesized compounds were confirmed using IR, NMR (^1^H and ^13^C) and mass spectral data, as well as reported values that are known. 

### 2.2. Biological Evaluation 

#### 2.2.1. Cytotoxicity

The cytotoxicity of the synthesized compounds **4a**–**k** was evaluated against two different cancer cell lines, namely MCF7 and A2780, and the results are summarized in Table 1. Among the tested compounds, the isatin-hydrazone **4j** exhibited the highest inhibitory activity against MCF7 cell lines (1.51 ± 0.09 µM). It should be noted that **4k** (3.56 ± 0.31), **4e** (5.46 ± 0.71), **4i** (7.77 ± 0.008) and **4f** (9.07 ± 0.59) showed moderate inhibitory activity against MCF7 cell lines. In the case of A2780 cell lines, however, only the halogen-substituted compounds **4e, 4j, 4k** and **4f** showed a little inhibitory activity. Nevertheless, all of the tested compounds were more sensitive towards MCF7 compared to A2780 cell lines.

**Figure 2:**
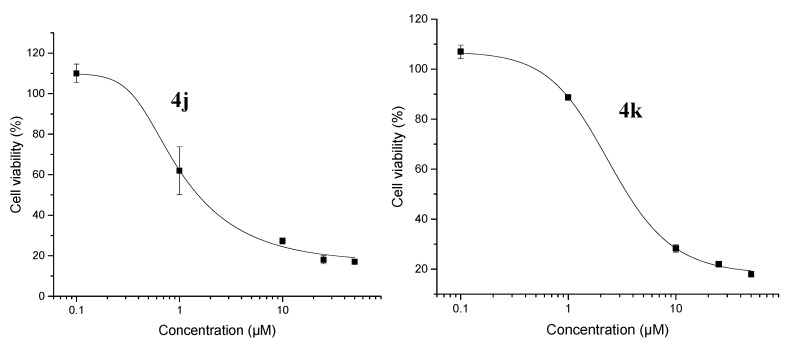
Dose–response curve of the most cytotoxic compounds against MCF7 cell lines.

Figure 2 shows the dose–response curves for compounds **4j** and **4k,** which were the most cytotoxic compounds against the breast cancer cells lines (MCF7) at a concentration of 1.51 and 3.56 µM, respectively. The IC_50_ values interpolated from dose–response data with five different concentrations were 0.1, 1, 10, 25 and 50 µM.

#### 2.2.2. Structure–Activity Relationships (SARs) Study of **4a**–**k**

**Figure 3:**
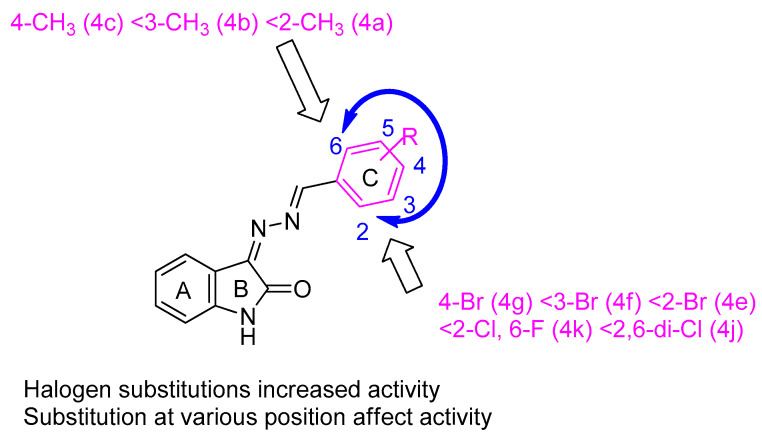
Structure–activity relationship (SAR) analysis of compounds **4a**–**k**.

The SARs study revealed that the cytotoxicity of **4a**–**k** increased or decreased in the same fashion as increases or decreases in halogen substitution in the aromatic C-ring. It is also related to the position of the substituents. As depicted in Figure 3, the bromo substituent at 4-position of the C-ring gave IC_50_ = 15.7 µM against MCF7 cell lines (Table 1, entry **4g**), while at 3-position, it increased to IC_50_ = 9.07 µM (Table 1, entry **4f**). Interestingly, the bromo substituent’s cytotoxicity at 2-position, increased dramatically to IC_50_ = 5.46 µM (Table 1, entry **4e**). Surprisingly, while 2- and 6-positions of the C-ring having respective chloro- and fluoro- substituents, the IC_50_ of compound **4k** was 3.56 µM (Table 1, entry **4k**). More surprisingly, with both 2- and 6-positions of the C-ring with chloro- substituents, compound **4j** exhibited the highest cytotoxicity of IC_50_ = 1.51 µM (Table 1, entry **4j**) which is two-fold more than the control anticancer drug doxorubicin (IC_50_ = 3.1 µM) (Table 1, entry doxorubicin). On the other hand, the methyl substituent at the C-ring also affects cytotoxicity against MCF7 cell lines. The methyl substituent at 4-position gave IC_50_ = 32.48 µM (Table 1, entry **4c**) and it increased at 3-position to IC_50_ = 14.65 µM (Table 1, entry **4b**), whereas at 2-position, the IC_50_ value was 10.82 µM (Table 1, entry **4a**). On the other hand, A2780 cell lines were inhibited by the halogenated derivatives **4e**, **4j**, **4k** and **4f**. In this case, 2-bromo substituted derivatives showed higher activity than the other three. 

#### 2.2.3. CDK2 Protein Kinase Inhibitory Activity of **4a**–**k**

The promising cytotoxicity of **4**, especially **4j** and **4k,** motivated us to study further inhibitory activities against CDK2 protein kinase. As summarized in Table 2, **4j** and **4k** exhibited good inhibitory activity against cyclin-dependent kinase 2 (CDK2), which is half of that of the known kinase inhibitor imatinib. 

### 2.3. In Silico Drug Likeness Property Analysis

Rational drug designing is the most significant part in modern drug discovery approaches. In this regard, computational ADME (absorption, distribution, metabolism and excretion) analysis can help us select the best drug in terms of cost, time and efficiency. Applying computational chemistry tools, in vitro and in vivo ADME prediction is now much more convenient and it can aid pharmaceutical industries to screen thousands of compounds within a short time [53]. Here, synthesized compounds (**4a**–**k**) were screened for predicted ADME values and the results are summarized in Table 3. Since high molecular weight compounds are always less effective in terms of intestinal absorption [54,55], our designed and synthesized isatin-hydrazones’ (**4a**–**k**) molecular weights were kept low, in between 263–328 Da. Compounds **4a**–**k** showed hydrogen bond donor (HBD) values of 1, except **4i** which had a HBD value of 2 (recommended value = ˂5), and hydrogen bond acceptor (HBA) values of 5, except **4i** which had HBA value = 6.5, **4h** with a HBA value = 5.75 and **4d** with **a** HBA value = 5.5 (recommended value = ˂10). On the other hand, doxorubicin (Doxo) showed a HBD value of 5 and HBA value of 15, which indicates that synthesized isatin-hydrazones are superior to Doxo in respect to HBD and HBA values. A parameter was established in 2002 to check the bioavailability of a drug using octanol/water partition coefficient and solubility scoring (recommended values for octanol/water partition coefficient are −2 – 6.5 and solubility scoring are −6.5 – 0.5 mol/dm−3) [56]. The octanol/water partition coefficient for hydrazones **4a**–**k** is in between 1.79–3.12 and solubility score is −3.39 – −4.35, respectively. Doxo showed a score within the reference values of −0.49 and −2.37, respectively. The hERG K^+^ channel blockers are potentially toxic for the heart, thus the recommended range for predicted logIC_50_ values for blockage of hERG K^+^ channels (loghERG) is > −5 [57]. Intriguingly, **4a**–**c** and **4e**–**k** showed higher values for loghERG score (> −5.58–−5.91) than Doxo (−6.02), except **4d,** which was similar to Doxo, which proved their (**4a**–**k)** toxicity to be lower than Doxo. The Caco-2 cell, considered as the reliable in vitro model to estimate oral drug absorption and transdermal delivery [58], was high (>1310) for all compounds except **4i** (487). Interestingly, Doxo had a much lower value (2.29) than **4a**–**k**, which signifies the improved oral drug absorption and transdermal delivery efficiency of the studied compounds compared to Doxo. 

The blood–brain barrier separates the CNS from blood, and a successful compound must pass into the blood stream, which depends on several factors, such as molecular weight, which must be below 480 [59]. Since our synthesized compounds have low molecular weights and fall within the recommended values, this, therefore, showed significant results. Madin–Darby canine kidney (MDCK) cell permeability is considered as the measurement of blood–brain barrier permeability, where greater than 500 is of great value and less than 25 indicates a very poor result according to Jorgensen’s rule of 3 [60]. Except compound **4i** (227), all the other compounds gave much higher MDCK values (> 666 to 3162) than Doxo (0.766 only). The synthesized compounds also gave a predicted human oral absorption rate of 100%, except compound **4i** which gave 86%. On the other hand, Doxo showed a predicted human oral absorption rate of 0%. Taken together, all the designed compounds, **4a**–**k**, of this study showed higher predicted ADME values than Doxo.

### 2.4. Architecture of the CDK2 Active Site

Developing new inhibitors against CDK2 mainly involves designing compounds that can act as ATP competitive inhibitors by binding to the ATP binding cleft of CDK2. According to the active and inactive state of the protein kinase, two different types of inhibitors can be designed: type I and type II inhibitors. Type I inhibitors mainly bind to the ATP binding pocket of an active kinase, whereas type II inhibitors bind to the inactive kinase [61]. From the recently published crystal structure of CDK2 in a complex with the inhibitor CVT-313, it was found that active kinase inhibition depends solely on the interaction with the DFG motif, which comprises Asp145-Phe146- Gly147. Leu83, Asp86 and Asp145 form the ATP binding site of CDK2 through hydrogen bonds, where Asp145 belongs to the DFG motif. Outside of the active site, the residues Glu81–Leu83 hinge linker sequence is responsible for flexibility of the kinase. The phosphorylation of the C-terminal domain contains the catalytic residue (Glu51) required for the phosphorylation of Thr160 in the T-loop for its activation. The activation segment is composed of the conserved DFG motif (Asp145-Phe146- Gly147) and the APE motif (Ala170-Pro171-Glu172). The unique PSTAIRE motif (Pro45–Glu51) in CDK2 that has a key role in its interaction with the cyclin subunit is found in the N-terminal domain [61]. To investigate whether the synthesized compounds (**4j** and **4k**, based on best cytotoxicity assay and enzyme inhibition assay against CDK2 protein kinase) are type I or type II inhibitors, and also to check their binding mechanism with CDK2, we performed a molecular docking analysis.

### 2.5. In Silico Binding Mechanism Analysis

**Figure 4:**
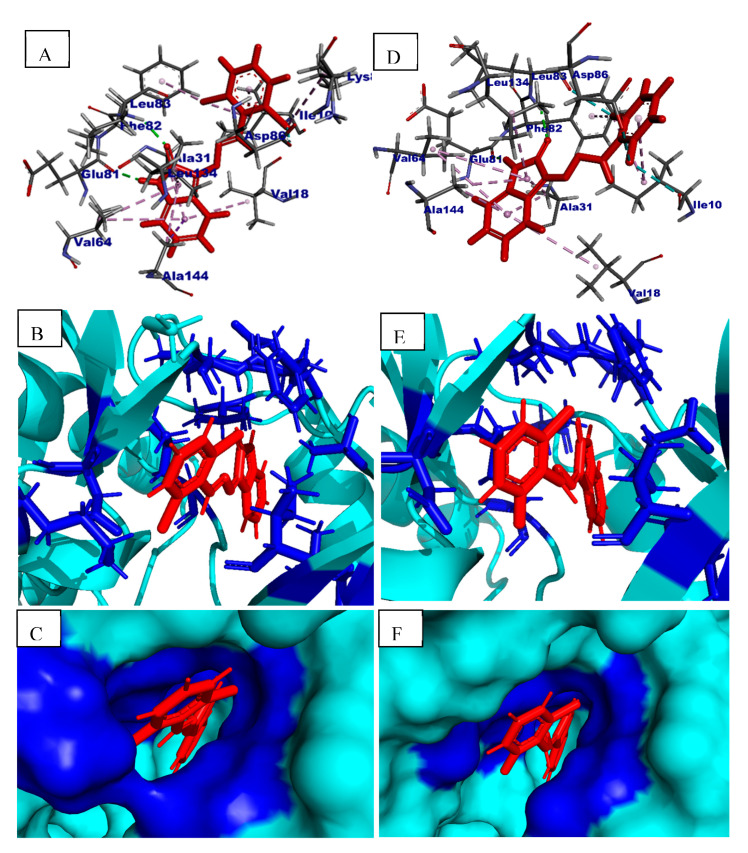
(**A**) 2D docking pose of **4j** within the active site of CDK2; (**B**) 3D docking pose of **4j** within the active site of CDK2; (**C**) binding pocket formed by interacting residues of active site of CDK2 surrounding **4j**; (**D**) 2D docking pose of **4k** within the active site of CDK2; (**E**) 3D docking pose of **4k** within the active site of CDK2; (**F**) binding pocket formed by interacting residues of active site of CDK2 surrounding **4k**. Compounds are shown in red color, protein in cyan and interacting residues in blue color.

From the docking analysis of compounds **4j** and **4k** with CDK2, it was clearly observed that both compounds interacted with the ATP binding pocket residues Leu83 and Asp86 but not with Asp145 of the DFG motif (Figure 4A,D). **4j** and **4k** thus can act as ATP competitive type II inhibitor by binding to inactive kinase [61]. Moreover, both the compounds showed a similar fashion of interactions, which involved several hydrogen bonds and ionic interactions with the binding site cavity surrounding residues, such as Ile10, Val18, Ala31, Val64, Glu81, Phe82, Leu134 and Ala144, which is consistent with the molecular docking analysis of 3,6-disubstituted pyridazines; 6-N,6-N-dimethyl-9-(2-phenylethyl)purine-2,6-diamine as CDK2 inhibitors (2, 3) [62,63]. However, compound **4j** formed one additional interaction with Lys89, which was absent in case of compound **4k**. It may bind to inactive kinase, which can be analyzed by finding no interaction between the compounds with catalytic residue Glu51 that is responsible for the phosphorylation of Thr160 for activation of kinase function. The interacting residues, although they do not belongs to the ATP binding pocket, formed a pathway for the compounds to bind properly to the ATP binding pocket. Glu81 to Leu83, on the other hand, forms the hinge region responsible for the flexibility of the protein kinase. From Figure 4C,F, it is visible how these residues form the binding cleft and pathway for the compounds to occupy the ATP binding site of CDK2 protein kinase. Figure 4B,E show the 3D interaction pattern and formation of binding cleft. From the docking analysis, it can be concluded that both **4j** and **4k** served as ATP competitive type II inhibitors by interacting with ATP binding pocket residues and with the residues that paved the way for the compounds to bind to the CDK2 ATP binding pocket. Table 4 summarizes the compounds and names the interacting residues, along with the types of interactions and the docking score of each compound.

## 3. Materials and Methods 

### 3.1. General 

Chemicals and solvents were of commercial reagent grade (Sigma-Aldrich, St. Louis, MO, USA) and were used without further purification. The progress of reactions and purity of reactants and products were checked using pre-coated silica gel 60 aluminum TLC sheets with fluorescent indicator UV254 of Macherey-Nagel, and detection was carried out with an ultraviolet light (254 nm) (Merck, Darmstadt, Germany). Melting points were measured using an Electrothermal IA9100 melting point apparatus (Stone, Stafforshire, ST15 OSA, UK). Infrared (IR) spectra (as KBr pellet) were recorded on a FT-IR Spectrum BX device from Perkin Elmer (Ayer Rajah Crescent, Singapore). ^1^H NMR spectra were recorded using a Bruker 600 MHz spectrometer (Reinstetten, Germany) and DMSO-d_6_ was used as a solvent. Chemical shifts were expressed in parts per million (ppm) relative to TMS as an internal standard. Mass spectra were taken with an Agilent 6410 Triple Quad mass spectrometer fitted with an electrospray ionization (ESI) ion source (Agilent Technologies, Palo Alto, CA, USA). 

### 3.2. (Z)-3-Hydrazonoindolin-2-one (***2***)

A mixture of isatin (0.1 mole) and hydrazine hydrate (1.2 equiv.) in methanol was refluxed for 1h and cooled to room temperature. The precipitate was filtered, washed with cold methanol and dried at room temperature in open air to give isatin monohydrazone in quantitative yield (~99%). A yellow powder was obtained (~99%). Mp. = 230–231 °C (Lit. [64] Mp. = 231–232 °C).

### 3.3. General Procedure for the Synthesis of 3-[benzylidene(substituted)hydrazono]indolin-2-ones **4a**–**k**

A mixture of isatin monohydrazone (**2**, 5 mmol) and 4-methylbenzaldehyde (**3a**, 5 mmol) in absolute ethanol (15 mL) was added to a few drops of glacial acetic acid. The reaction mixture was refluxed for 4 h. The completion of the reaction was monitored by TLC. The precipitate solid was filtered, washed with cold ethanol and air dried, and was then further purified by recrystallization using ethanol, obtained **4a** as yellow powder. Please see in Supplementary Materials for NMR (^1^H & ^13^C) and MS spectra of compound **4** (in Supplementary Materials).

#### 3.3.1. 3-((2-Methylbenzylidene)hydrazono)indolin-2-one (**4a**)

Yellow powder (80%). Mp. = 198–199 °C. IR (KBr) ν_max_(cm^−1^): 3238 (N-H), 2910 (C-H), 1728 (C=O), 1612 (C=N). ^1^H NMR (DMSO-d_6_, 600 MHz) (ppm), δ 2.52 (s, 3H, -CH_3_), 6.89 (t, 1H, ArH), 7.01 (t, 1H, ArH), 7.32–7.44 (m, 4H, ArH), 7.89 (t, 1H, ArH), 8.06 (t, 1H, ArH), 8.80 (s, 1H), 10.88 (s, 1H, -NH). ^13^C NMR (DMSO-d_6_, 150 MHz) (ppm), δ 165.02, 159.58, 150.71, 145.45, 139.72, 134.20, 132.31, 131.78, 129.00, 127.96, 127.06, 122.83, 116.81, 111.35 and 19.58. ESI mass *m/z =* 264 [M + H]^+^; 286 [M + Na]^+^.

#### 3.3.2. 3-((3-Methylbenzylidene)hydrazono)indolin-2-one (**4b**)

Yellow powder (82%). Mp. = 183–184 °C. ^1^H NMR (DMSO-d_6_, 600 MHz) (ppm), δ 2.39 (s, 3H, -CH_3_), 6.89 (t, 1H, ArH), 7.02 (t, 1H, ArH), 7.39 (m, 2H, ArH). 7.44 (t, 1H, ArH), 7.56 (m, 2H, ArH), 7.88 (t, 1H, ArH), 8.53 (s, 1H), 10.86 (s, 1H, -NH). ^13^C NMR (DMSO-d_6_, 150 MHz) (ppm), δ 164.93, 160.61, 150.64, 145.46, 139.02, 134.19, 133.83, 133.28, 129.87, 129.58, 129.20, 126.34, 122.86, 116.82, 111.32 and 21.36. ESI mass *m/z =* 264 [M + H]^+^; 286 [M + Na]^+^. 

#### 3.3.3. 3-((4-Methylbenzylidene)hydrazono)indolin-2-one (**4c**)

Orange powder (75%). Mp. = 230–231 °C. (Lit. [65] mp. = 231 °C) IR (KBr) ν_max_(cm^−1^): 3182 (N-H), 2839 (C-H), 1716 (C=O), 1612 (C=N). ^1^H NMR (DMSO-d_6_, 600 MHz) (ppm), δ 2.38 (s, 3H, -CH_3_), 6.88 (t, 1H, ArH), 7.02 (t, 1H, ArH), 7.37 (m, 3H, ArH), 7.86 (m, 2H, ArH), 7.93 (t, 1H, ArH), 8.58 (s, 1H), 10.86 (s, 1H, -NH). ^13^C NMR (DMSO-d_6_, 150 MHz) (ppm), δ 165.02, 161.34, 150.91, 145.4, 142.96, 134.11, 131.29, 130.3, 129.39, 129.26, 122.83, 116.91, 111.28 and 21.73. ESI mass *m/z =* 264 [M + H]^+^; 286 [M + Na]^+^.

#### 3.3.4. 3-((4-(Methylthio)benzylidene)hydrazono)indolin-2-one (**4d**)

Red crystals (79%). Mp. = 204–205 °C. IR (KBr) ν_max_(cm^−1^): 3278 (N-H), 2920 (C-H), 1732 (C=O), 1612 (C=N). ^1^H NMR (DMSO-d_6_, 600 MHz) (ppm), δ 2.53 (s, 3H, S-CH_3_), 6.88 (t, 1H, ArH), 7.02 (t, 1H, ArH), 7.33–7.50 (m, 3H, ArH). 7.77–7.95 (m, 3H, ArH). 8.59 (s, 1H), 10.84 (s, 1H, -NH). ^13^C NMR (DMSO-d_6_, 150 MHz) (ppm), δ 165.07, 161.47, 151.01, 145.40, 144.69, 134.09, 130.09, 129.75, 129.28, 129.13, 126.05, 125.93, 122.79, 116.97, 111.27 and 14.50. ESI mass *m/z =* 296 [M + H]^+^; 318 [M + Na]^+^. 

#### 3.3.5. 3-((2-Bromobenzylidene)hydrazono)indolin-2-one (**4e**)

Yellow powder (93%). Mp. = 233–234 °C. IR (KBr) ν_max_(cm^−1^): 3194 (N-H), 2818 (C-H), 1730 (C=O), 1535 (C=N). ^1^H NMR (DMSO-d_6_, 600 MHz) (ppm), δ 6.89 (d, *J*=1.2 Hz, 1H, ArH), 7.02 (t, 1H, ArH), 7.40 (t, 1H, ArH), 7.51 (t, 1H, ArH), 7.59 (t, 1H, ArH), 7.80–7.58 (m, 2H, ArH), 7.22 (t, 1H, ArH), 8.72 (s, 1H), 10.90 (s, 1H, -NH). ^13^C NMR (DMSO-d_6_, 150 MHz) (ppm), δ 164.75, 158.16, 150.99, 145.73, 134.56, 134.18, 134.13, 132.23, 129.25, 129.09, 125.60, 122.93, 116.62 and 111.45. ESI mass *m/z =* 328 [M(^79^Br) + H]^+^, 330 [M(^81^Br) + H]^+^; 350 [M(^79^Br) + Na]^+^, 352 [M(^81^Br) + Na]^+^. 

#### 3.3.6. 3-((3-Bromobenzylidene)hydrazono)indolin-2-one (**4f**)

Yellowish brown powder (92%). Mp. = 182–183 °C. IR (KBr) ν_max_(cm^−1^): 3412 (N-H), 2920 (C-H), 1714 (C=O), 1676 (C=N). ^1^H NMR (DMSO-d_6_, 600 MHz) (ppm), δ 6.89 (t, 1H, ArH), 7.01 (t, 1H, ArH), 7.39–7.53 (m, 2H, ArH), 7.71–7.87 (m, 2H, ArH), 7.99–8.10 (m, 1H, ArH), 8.54 (s, 1H), 10.91 (s, 1H, -NH). ^13^C NMR (DMSO-d_6_, 150 MHz) (ppm), δ 164.77, 161.07, 158.28, 150.52, 145.60, 136.16, 134.98, 134.42, 131.97, 131.84, 131.60, 131324, 129.16, 127.72, 127.56, 122.92, 116.64 and 111.41. ESI mass *m/z =* 328 [M(^79^Br) + H]^+^, 330 [M(^81^Br) + H]^+^; 350 [M(^79^Br) + Na]^+^, 352 [M(^81^Br) + Na]^+^. 

#### 3.3.7. 3-((4-Bromobenzylidene)hydrazono)indolin-2-one (**4g**)

Orange powder (90%). Mp. = 267–268 °C. IR (KBr) ν_max_(cm^−1^): 3169 (N-H), 2879 (C-H), 1735 (C=O), 1616 (C=N). ^1^H NMR (DMSO-d_6_, 600 MHz) (ppm), δ 6.89 (t, 1H, ArH), 7.01 (t, 1H, ArH), 7.39 (t, 1H, ArH), 7.77 (t, 2H, ArH), 7.83 (t, 1H, ArH), 7.90 (t, 2H, ArH), 8.58 (s, 1H), 10.89 (s, 1H, -NH). ^13^C NMR (DMSO-d_6_, 150 MHz) (ppm), δ 164.87, 159.42, 150.73, 145.52, 134.35, 133.01, 132.75, 131.05, 129.25, 126.18, 122.90, 116.72 and 111.38. ESI mass *m/z =* 328 [M(^79^Br) + H]^+^, 330 [M(^81^Br) + H]^+^; 350 [M(^79^Br) + Na]^+^, 352 [M(^81^Br) + Na]^+^. 

#### 3.3.8. 3-((4-Methoxy-2,6-dimethylbenzylidene)hydrazono)indolin-2-one (**4h**)

Orange powder (90%). Mp. = 251–252 °C. IR (KBr) ν_max_(cm^-1^): 3182 (N-H), 2839 (C-H), 1716 (C=O), 1612 (C=N). ^1^H NMR (DMSO-d_6_, 600 MHz) (ppm), δ 2.18 (s, 3H, -CH_3_) 2.52 (s, 3H, -CH_3_) 3.85 (t, 3H, OCH_3_), 6.88 (d, *J* = 9, 2H, ArH), 7.03 (t, 1H, ArH), 7.36 (t, 1H, ArH), 7.85 (t, 1H, ArH), 8.02 (t, 1H, ArH), 8.78 (s, 1H), 10.82 (s, 1H, -NH). ^13^C NMR (DMSO-d_6_, 150 MHz) (ppm), δ 165.32, 161.75, 160.97, 150.83, 145.19, 140.40, 133.81, 130.36, 129.01, 124.59, 123.77, 122.79, 117.04, 113.28, 111.17, 56.06, 19.70 and 16.19. ESI mass *m/z =* 308 [M + H]^+^; 330 [M + Na]^+^. 

#### 3.3.9. 3-((2-Hydroxy-4-methoxybenzylidene)hydrazono)indolin-2-one (**4i**)

Reddish brown (98%). Mp. = 242–243 °C. IR (KBr) ν_max_(cm^−1^): 3188 (O-H), 2910 (C-H), 1724 (C=O), 1620 (C=N). ^1^H NMR (DMSO-d_6_, 600 MHz) (ppm), δ 3.81 (s, 3H, OCH_3_), 6.52 (t, 1H, ArH), 6.59 (t, 1H, ArH), 6.87 (t, 1H, ArH), 7.04 (t, 1H, ArH), 7.40 (t, 1H, ArH), 7.55 (t, 1H, ArH), 7.60 (t, 1H, ArH), 8.97 (s, 1H), 10.9 (s, 1H, -NH), 12.31 (s, 1H, -OH). ^13^C NMR (DMSO-d_6_, 150 MHz) (ppm), δ 167.89, 165.07, 163.03, 159.79, 150.21, 144.64, 135.36, 133.90, 122.69, 120.40, 111.69, 111.20, 108.06, 101.54 and 56.08. ESI mass *m/z =* 296 [M + H]^+^; 318 [M + Na]^+^. 

#### 3.3.10. 3-((2,6-Dichlorobenzylidene)hydrazono)indolin-2-one (**4j**)

Orange powder (98%). Mp. = 286–287 °C. IR (KBr) ν_max_(cm^−1^): 3165 (N-H), 2812 (C-H), 1730 (C=O), 1618 (C=N). ^1^H NMR (DMSO-d_6_, 600 MHz) (ppm), δ 6.89 (t, 1H, ArH), 6.97 (t, 1H, ArH), 7.39–7.55 (m, 2H, ArH), 7.65 (t, 2H, ArH), 7.83 (t, 1H, ArH), 8.71 (s, 1H), 10.91 (s, 1H, -NH). ^13^C NMR (DMSO-d_6_, 150 MHz) (ppm), δ 164.67, 155.50, 150.49, 145.80, 134.76, 134.72, 133.03, 129.98, 128.85, 122.77, 116.48 and 111.46. ESI mass *m/z =* 318 [M(^35^Cl) + H]^+^, 320 [M(^37^Cl) + H]^+^; 340 [M(^35^Cl) + Na]^+^, 342 [M(^37^Cl) + Na]^+^. 

#### 3.3.11. 3-((2-Chloro-6-fluorobenzylidene)hydrazono)indolin-2-one (**4k**)

Reddish brown (75%). Mp. = 277–778 °C. IR (KBr) ν_max_(cm^−1^): 3165 (N-H), 2852 (C-H), 1732 (C=O), 1620 (C=N). ^1^H NMR (DMSO-d_6_, 600 MHz) (ppm), δ 6.89 (t, 1H, ArH), 6.99 (t, 1H, ArH), 7.39–7.45 (m, 2H, ArH), 7.52 (t, 1H, ArH), 7.62 (t, 1H, ArH), 7.94 (t, 1H, ArH), 8.73 (s, 1H), 10.90 (s, 1H, -NH). ^13^C NMR (DMSO-d_6_, 150 MHz) (ppm), δ 164.80, 162.18, 160.46, 154.25, 150.99, 145.77, 135.57, 134.74, 134.35, 134.29, 128.86, 127.07, 122.78, 119.84, 119.76, 116.67, 116.58, 116.44 and 111.43. ESI mass *m/z =* 302 [M(^35^Cl) + H]^+^, 304 [M(^37^Cl) + H]^+^ 324 [M(^35^Cl) + H]^+^, 326 [M(^37^Cl) + H]^+^. 

### 3.4. Cytotoxicity

The cytotoxicity of the synthesized compounds was evaluated by MTT assay, as previously described [66]. Two cancer cell lines, MCF7 (human breast adenocarcinoma) and A2780 (human ovary adenocarcinoma), were used in this study, which were obtained from the ATCC (Rockville, MD, USA). They were sub-cultured in RPMI-1640 media (supplemented with 10% FBS and 1% antibiotics) at 37 °C and 5% CO_2_. Additionally, compounds were prepared at the same medium to obtain serial dilutions (50, 25, 19,1 and 0.1 µM). The two cell lines were separately cultured in 96-well plates (3 × 10^3^/well) and incubated at 37 °C overnight. The following day, before treating the cells with the compounds, each well of the T0 plate was treated with 50 μL MTT solution (2 mg/mL in phosphate buffered saline) and then incubated for 2–4 h. The media were aspirated, and the formazan crystals were solubilized by adding 150 µL DMSO. Absorbance was read on a multi-plate reader (BioRad) at 550 mm. Optical density of the purple formazan A550 was proportional to the number of viable cells. Compound concentration causing 50% inhibition (IC_50_) compared to control cell growth (100%) was determined. The data were obtained from triplicates and analyzed using statistical software.

### 3.5. In Vitro Cyclin Dependent Kinase2 (CDK2) Inhibitory Activity 

The CDK2 Assay Kit is designed to measure CDK2/CyclinA2 activity for screening and profiling applications, using Kinase-Glo^®^ MAX as a detection reagent. The CDK2 Assay Kit comes in a convenient 96-well format, with enough purified recombinant CDK2/CyclinA2 enzyme, CDK substrate peptide, ATP and kinase assay buffer for 100 enzyme reactions [67]. The assay was performed according to the protocol supplied from the CDK2 Assay kit #79599. The CDK2/CyclinA2 activity at a single dose concentration of 10μM was performed, where the Kinase-Glo MAX luminescence kinase assay kit (Promega#V6071) was used. The compounds were diluted in 10% DMSO and 5 μL of the dilution was added to a 50 μL reaction so that the final concentration of DMSO was 1% in all of the reactions. All of the enzymatic reactions were conducted at 30 °C for 40 min. The 50 μL reaction mixture contained 40 mM Tris, pH 7.4, 10 mM MgCl_2_, 0.1 mg/mL BSA, 1 mM DTT, 10 mM ATP, Kinase substrate and the enzyme (CDK2/CyclinA2). After the enzymatic reaction, 50 μL of Kinase-Glo^®^ MAX Luminescence kinase assay solution was added to each reaction and the plates were incubated for 5 min at room temperature. Luminescence signal was measured using a Bio Tek Synergy 2 microplate reader.

### 3.6. Molecular Docking and In-Silico ADME Analysis

For molecular docking purposes, the Protein Data Bank (PDB) structure corresponding to the CDK2 protein kinase was downloaded from the Research Collaboratory for Structural Bioinformatics (RCSB) PDB database (https://www.rcsb.org/) in PDB format. The PDB ID used for CDK2 protein kinase was 2BHY. Proteins and compounds were prepared for docking by using an established procedure [68]. Discovery Studio was used for making 2D interaction figures. Pymol was used to generate the 3D and surface representation figures. For the in silico ADME analysis, all the compounds’ structures were prepared with the LigPrep module of Schrodinger Maestro and ADME was calculated by the Qikprop module of the same software package [69]. 

## 4. Conclusions

A series of novel isatin-hydrazones (**4a**–**b and 4d**–**k**), with a known compound **4c,** were designed and synthesized with good to moderate yields for cytotoxicity evaluation for the development of potent anticancer therapeutics. Among the compounds, **4j** showed a two-fold increase in cytotoxicity compared to the known cancer drug doxorubicin, and **4k** showed a similar cytotoxicity. The IC_50_ value of compound **4j** was 1.51 and for **4k** it was 3.56 µM, whereas doxorubicin had a 3.1 µM concentration against human breast adenocarcinoma (MCF7) cell lines. The most active compounds, **4j** and **4k,** were further evaluated for their inhibitory activities against CDK2 protein kinase. As expected, **4j** and **4k** exhibited good inhibitory activity against cyclin-dependent kinase 2 (CDK2) 0.2456 and 0.3006 µM, respectively, which is comparable to kinase imatinib 0.1512 µM. Highly recommended predicted ADME values were obtained than the known doxorubicin. The molecular docking study of **4j** and **4k** with CDK2 protein kinase revealed that they interacted with ATP binding pocket residues and lacked interactions with the active state DFG motif residues; therefore, **4j** and **4k** can be considered as ATP competitive type II inhibitors against CDK2 protein kinase. In conclusion, these simple molecules, isatin-hydrazones **4j** and **4k,** can be used as potential agents for anticancer therapeutics in further mechanism and toxicity studies. 

## Figures and Tables

**Table 1:** Cytotoxicity of **4a**–**k** against MCF7 and A2780 cell lines.

Compound	IC_50_ (µM)	
	MCF7	A2780
**4a**	10.82 ± 0.05	>50
**4b**	14 ± 1.33	>50
**4c**	32.48 ± 0.52	>50
**4d**	24 ± 2.61	>50
**4e**	5.46 ± 0.71	19 ± 2.52
**4f**	9.07 ± 0.59	25 ± 2.82
**4g**	15.70 ± 0.78	>50
**4h**	25.78 ± 0.13	>50
**4i**	7.77 ± 0.008	>50
**4j**	1.51 ± 0.09	26 ± 2.24
**4k**	3.56 ± 0.31	27 ± 3.20
Doxorubicin	3.10 ± 0.29	0.20 ± 0.03

**Table 2:** Inhibitory activities of compounds **4j** and **4k** against CDK2 protein kinase.

Compounds	CDK2 Protein Kinase (IC_50_ in µM) *
**4j**	0.2456
**4k**	0.3006
**Imatinib**	0.1312

* IC_50_ values are the mean ± SD of triplicate measurements.

**Table 3:** Analysis of drug likeness and pharmacokinetic properties by QikProp for compounds **4a–k**.

No.	MW ^a^	HBD ^b^	HBA ^c^	logPo/w ^d^	logS ^e^	logP	HERG ^f^	Caco-2 ^g^	BBB ^h^	MDCK ^i^	HOA(%) ^j^
**4a**	263	1	5	2.59	−3.82	9.8	−5.84	1324	−0.49	670	100
**4b**	263	1	5	2.62	−3.95	9.7	−5.89	1317	−0.51	666	100
**4c**	263	1	5	2.62	−3.95	9.7	−5.89	1317	−0.51	666	100
**4d**	295	1	5.5	2.93	−4.31	10.0	-6.03	1310	−0.49	1126	100
**4e**	328	1	5	2.83	−4.06	9.9	−5.90	1323	−0.33	1584	100
**4f**	328	1	5	2.88	−4.19	9.8	−5.91	1317	−0.32	1766	100
**4g**	328	1	5	2.88	−4.19	9.8	−5.91	1317	−0.32	1766	100
**4h**	307	1	5.75	2.89	−4.14	9.8	−5.58	1760	−0.44	911	100
**4i**	295	2	6.5	1.79	−3.39	12.4	−5.74	487	−1.07	227	86
**4j**	318	1	5	3.12	−4.35	9.6	−5.67	1616	−0.13	3162	100
**4k**	302	1	5	2.90	−4.17	9.7	−5.75	1432	−0.24	2158	100
**Doxo ^k^**	544	5	15	-0.49	−2.37	24.2	−6.02	2.29	−2.95	0.766	0

^a^ Molecular weight in Daltons (acceptable range: <500); ^b^ hydrogen bond donor (acceptable range: ≤5); ^c^ hydrogen bond acceptor (acceptable range: ≤10); ^d^ predicted octanol/water partition coefficient (acceptable range: −2–6.5); ^e^ predicted aqueous solubility, S in mol/dm−3 (acceptable range: −6.5–0.5); ^f^ predicted IC_50_ value for blockage of hERG K+ channels (concern: below −5); ^g^ Caco−2 value, permeability to Caco−2 (human colorectal carcinoma) cells in vitro; ^h^ blood−brain barrier permeability (acceptable range: ~−0.4); ^i^ predicted apparent Madin–Darby canine kidney (MDCK) cell permeability in nm/sec, QPPMDCK= >500 is great, <25 is poor; ^j^ predicted human oral absorption on 0% to 100% scale (<25% is poor and >80% is high); ^k^ Doxo = Doxorubicin.

**Table 4:** Docking score, interacting residues and types of interaction mediated by **4j** and **4k** with the ATP binding pocket of CDK2 protein kinase.

Compounds	Docking Score	Interacting Residues	Types of Interaction
**4j**	−6.5	Ile10, Val18, Ala31, Val64, Glu81, Phe82, Leu83, Asp86, Lys89, Leu134 and Ala144	Hydrogen π-Alkyl Halogen π-σ
**4k**	−5.9	Ile10, Val18, Ala31, Val64, Glu81, Phe82, Leu83, Asp86, Leu134 and Ala144	Hydrogen σ-Alkyl Halogen

## Supplementary Materials

The following are available online, Figure S1.: Proton (^1^H) Spectra of **4a**, Figure S2.: Carbon (^13^C) Spectra of **4a,** Figure S3.: Proton (^1^H) Spectra of **4g**, Figure S4.: Carbon (^13^C) Spectra of **4g,** Figure S5.: Proton (^1^H) Spectra of **4h**, Figure S6.: Carbon (^13^C) Spectra of **4h,** Figure S7.: Proton (^1^H) Spectra of **4i**, Figure S8.: Carbon (^13^C) Spectra of **4i,** Figure S9.: Proton (^1^H) Spectra of **4j**, Figure S10.: Carbon (^13^C) Spectra of **4j,** Figure S11.: Proton (^1^H) Spectra of **4k**, Figure S12.: Carbon (^13^C) Spectra of **4k,** Figure S13.: Mass Spectra of **4a**, Figure S14.: Mass Spectra of **4b**, Figure S15.: Mass Spectra of **4c**, Figure S16.: Mass Spectra of **4d**, Figure S17.: Mass Spectra of **4e**, Figure S18.: Mass Spectra of **4f**, Figure S19.: Mass Spectra of **4g**, Figure S20.: Mass Spectra of **4g**, Figure S21.: Mass Spectra of **4i**, Figure S22.: Mass Spectra of **4j**, Fifure S23.: Mass Spectra of **4k**.
