# Supplementary material for: A Series of Isatin-Hydrazones with Cytotoxic Activity and CDK2 Kinase Inhibitory Activity: A Potential Type II ATP Competitive Inhibitor
Source: Molecules. 2020 Sep 25;25(19):4400. doi: 10.3390/molecules25194400 (PMC7582667; doi:10.3390/molecules25194400)
Supplement: Supplementary file 1 [file molecules-25-04400-s001.pdf]

A Series of Isatin-Hydrazones with Cytotoxic Activity and CDK2 Kinase Inhibitory Activity: A Potential Type II ATP Competitive Inhibitor

Huda S. Al-Salem [1,*], Md Arifuzzaman [2], Hamad M. Alkahtani [1], Ashraf N. Abdalla [3], Iman S. Issa [1], Aljawharah Alqathama [4], Fatemah S. Albalawi [1] and A. F. M. Motiur Rahman [1,*]

[1]Department of Pharmaceutical Chemistry, College of Pharmacy, King Saud University, Riyadh 11451, Saudi Arabia;
[2]Yeungnam University, Gyeongsan 38541, Korea;
[3]Department of Pharmacology and Toxicology, Faculty of Pharmacy, Umm Al-Qura University, Makkah 21955, Saudi Arabia;
[4]Department of Pharmacognosy, Faculty of Pharmacy, Umm Al-Qura University, Makkah 21955, Saudi Arabia;

*****Correspondence: hhalsalem@ksu.edu.sa (H.S.A.-S.); afmrahman@ksu.edu.sa (A.F.M.M.R.); Tel.: +966-11-29-52740 (H.S.A.); +966-11-46-70237 (A.F.M.M.R.)

Entry	Content	Page no.	Entry	Content	Page no.
Figure S1	Proton (^{1}H) Spectra of **4a**	3	Figure S13	Mass Spectra of **4a**	15
Figure S2	Carbon (^{13}C) Spectra of **4a**	4	Figure S14	Mass Spectra of **4b**	16
Figure S3	Proton (^{1}H) Spectra of **4g**	5	Figure S15	Mass Spectra of **4c**	17
Figure S4	Carbon (^{13}C) Spectra of **4g**	6	Figure S16	Mass Spectra of **4d**	18
Figure S5	Proton (^{1}H) Spectra of **4h**	7	Figure S17	Mass Spectra of **4e**	19
Figure S6	Carbon (^{13}C) Spectra of **4h**	8	Figure S18	Mass Spectra of **4f**	20
Figure S7	Proton (^{1}H) Spectra of **4i**	9	Figure S19	Mass Spectra of **4g**	21
Figure S8	Carbon (^{13}C) Spectra of **4i**	10	Figure S20	Mass Spectra of **4h**	22
Figure S9	Proton (^{1}H) Spectra of **4j**	11	Figure S21	Mass Spectra of **4i**	23
Figure S10	Carbon (^{13}C) Spectra of **4j**	12	Figure S22	Mass Spectra of **4j**	24
Figure S11	Proton (^{1}H) Spectra of **4k**	13	Figure S23	Mass Spectra of **4k**	25
Figure S12	Carbon (^{13}C) Spectra of **4k**	14			

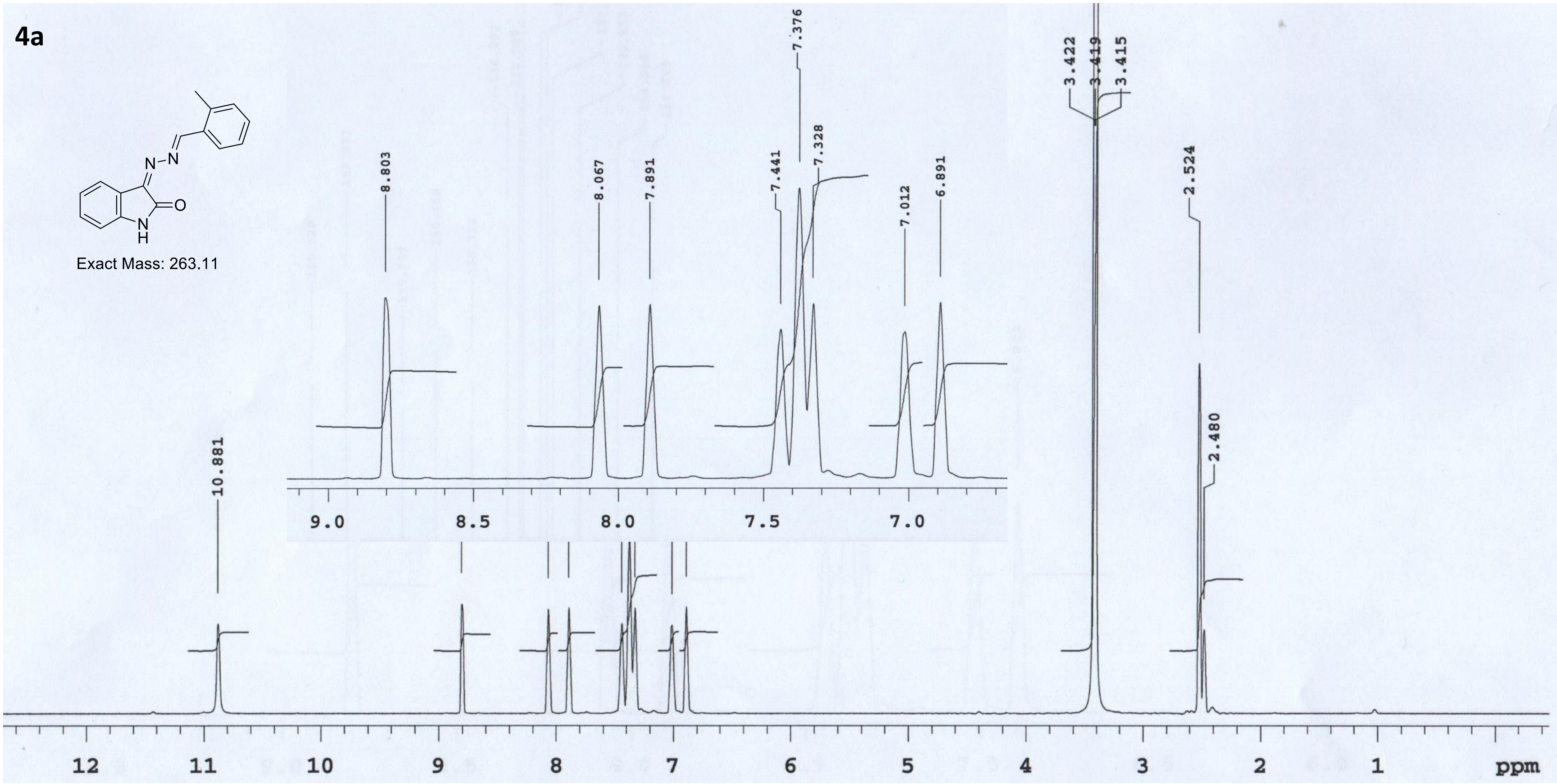

Figure S1. Proton (^{1}H) Spectra of **4a**

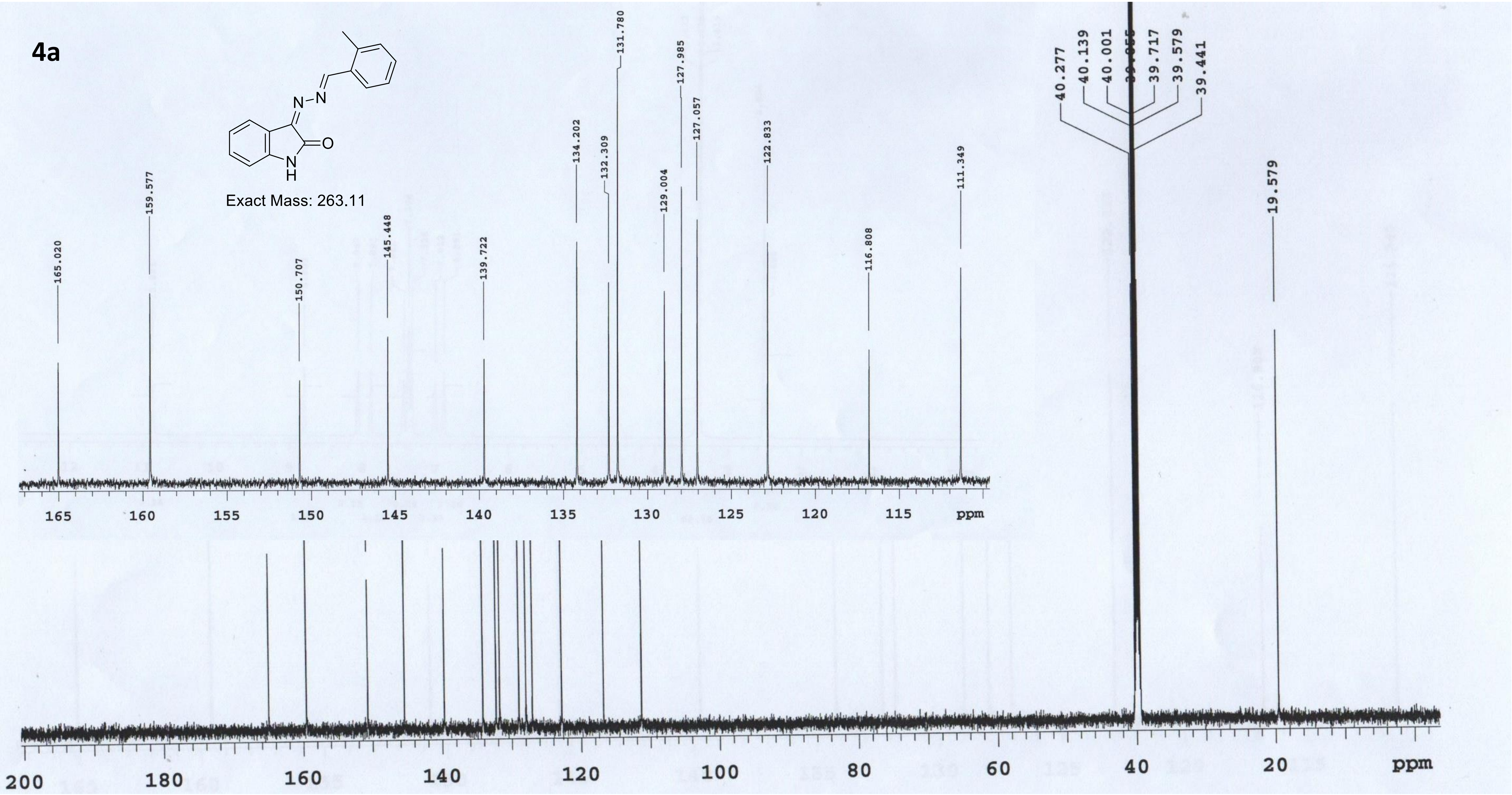

Figure S2. Carbon (^{13}C) Spectra of **4a**

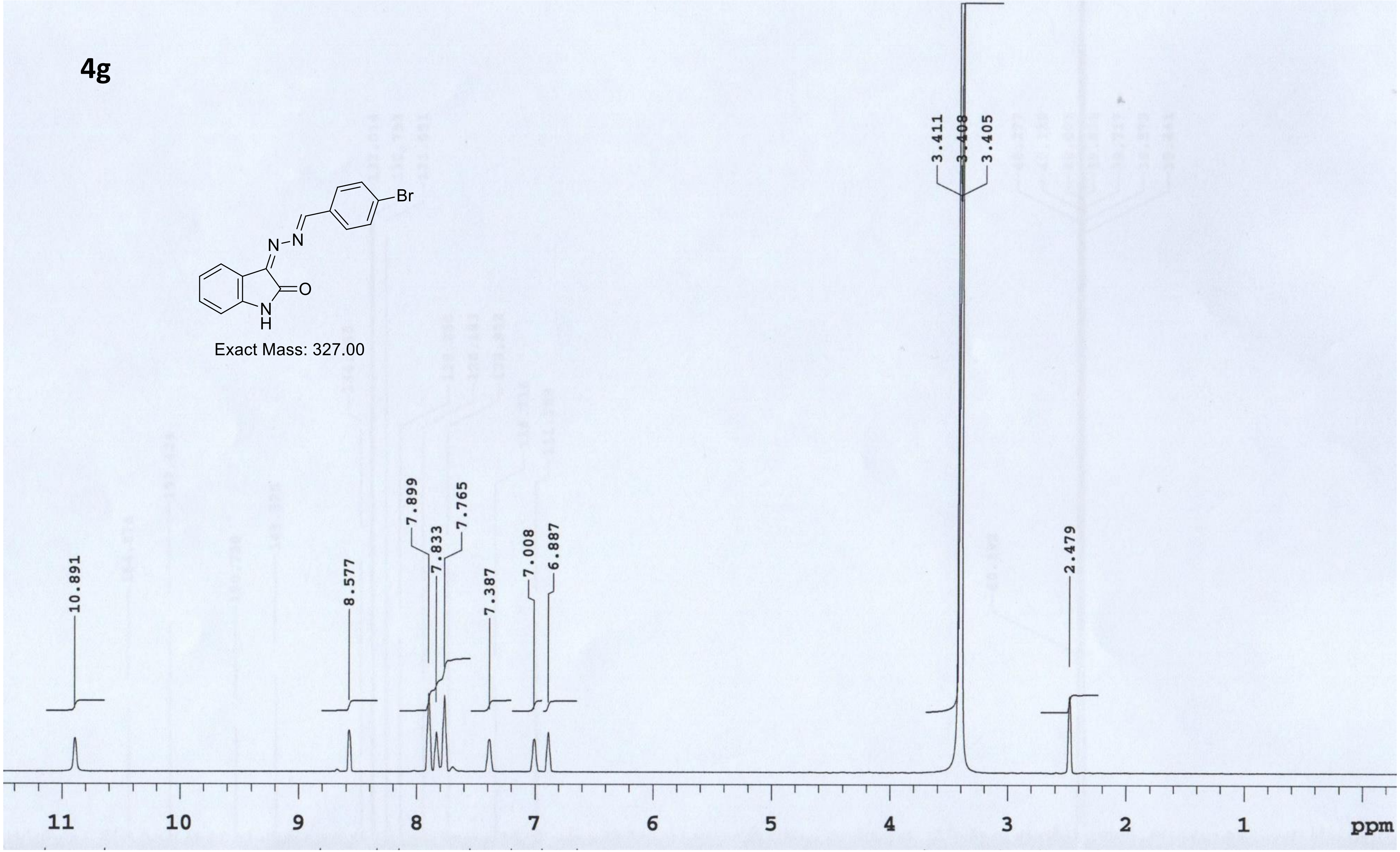

Figure S3. Proton (^{1}H) Spectra of **4g**

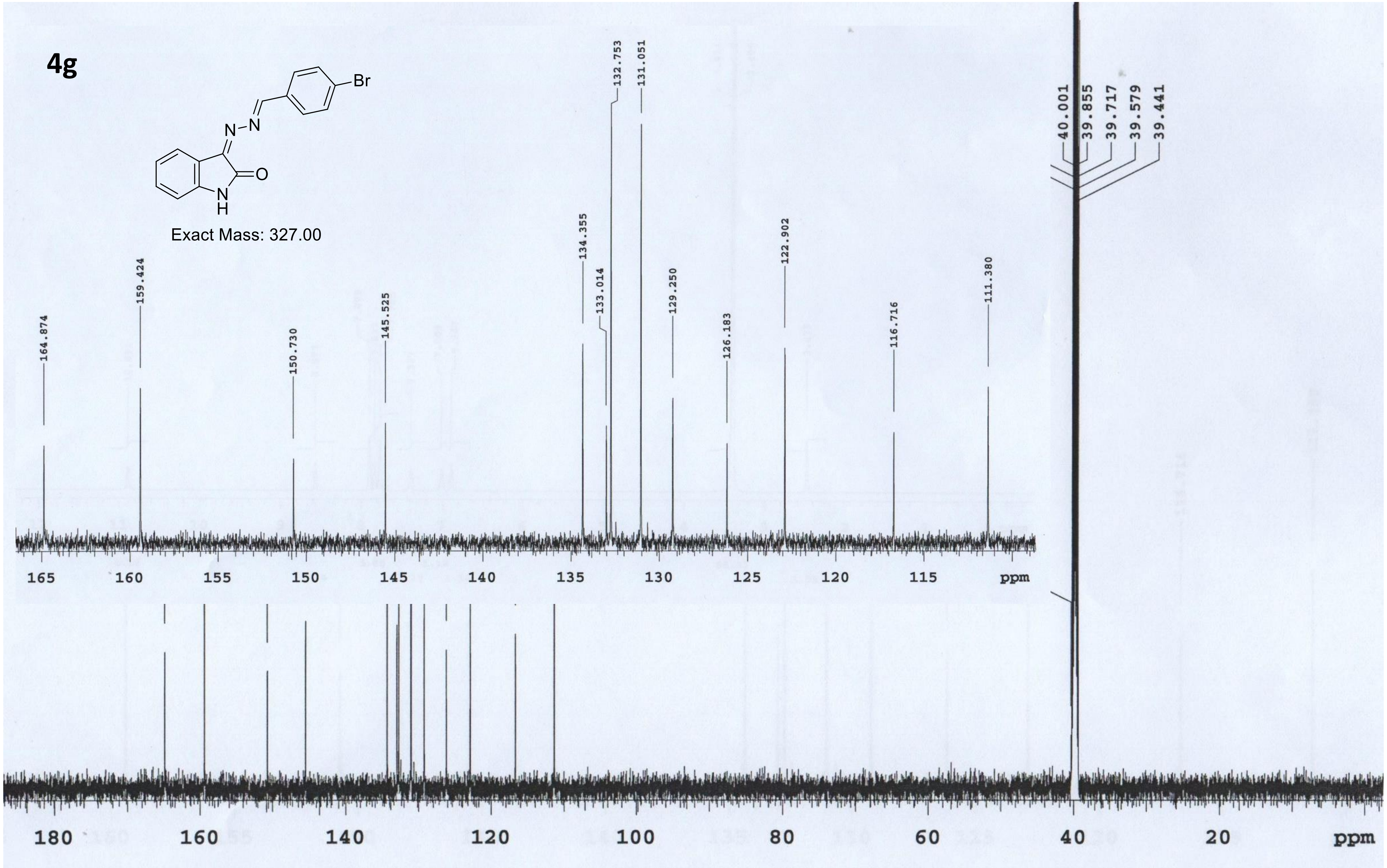

Figure S4. Carbon (^{13}C) Spectra of **4g**

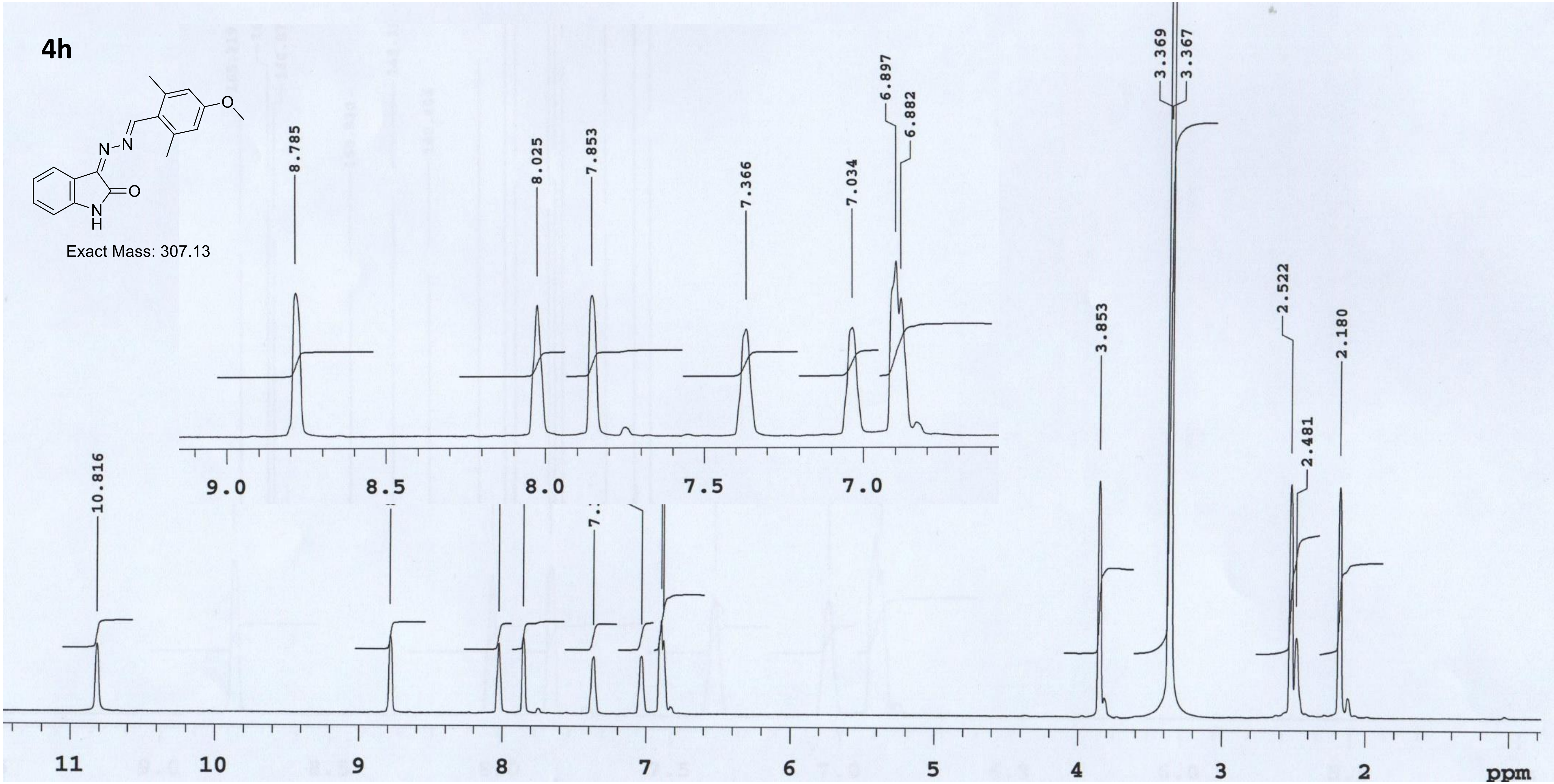

Figure S5. Proton (^{1}H) Spectra of **4h**

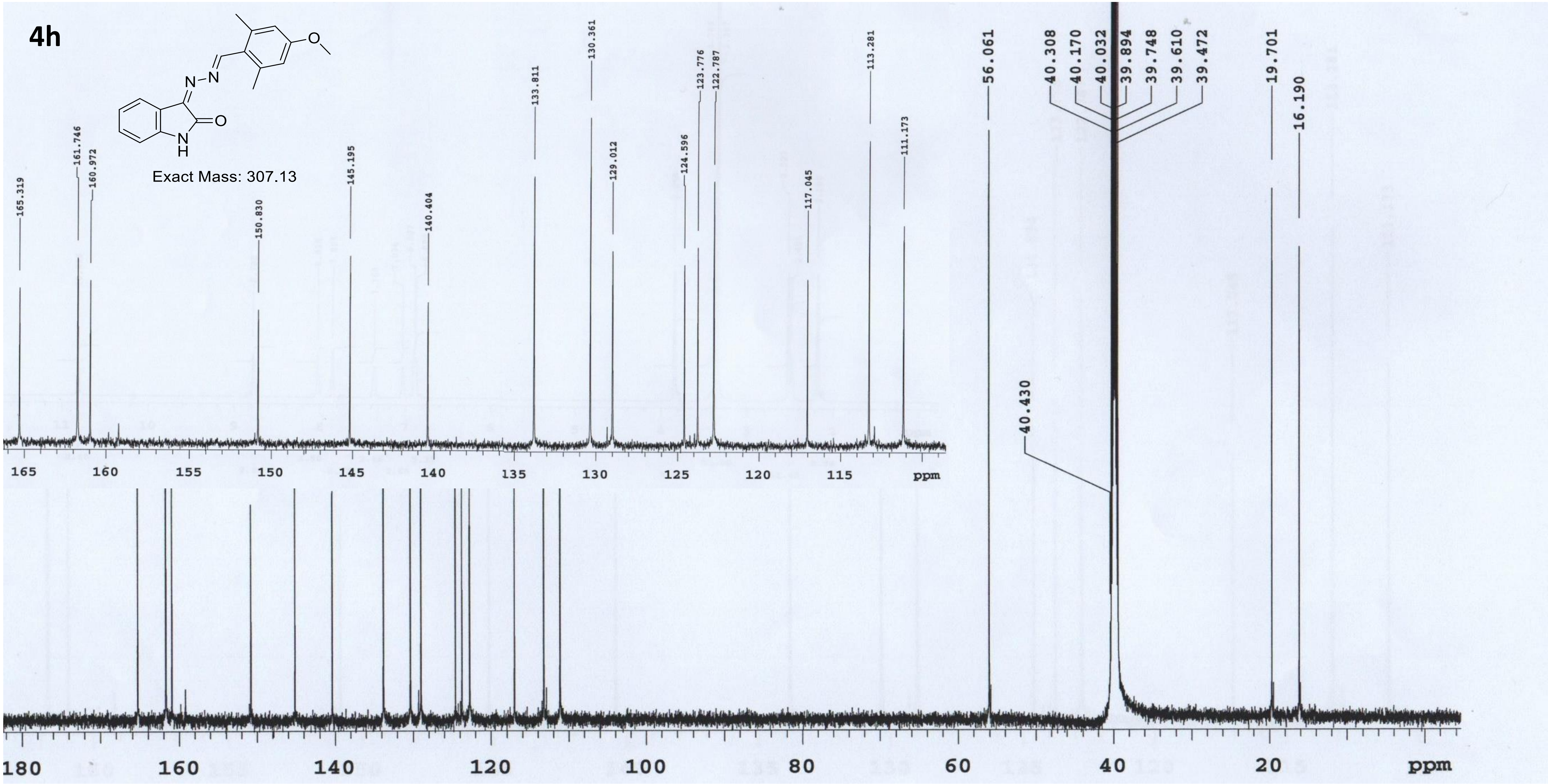

Figure S6. Carbon (^{13}C) Spectra of **46**

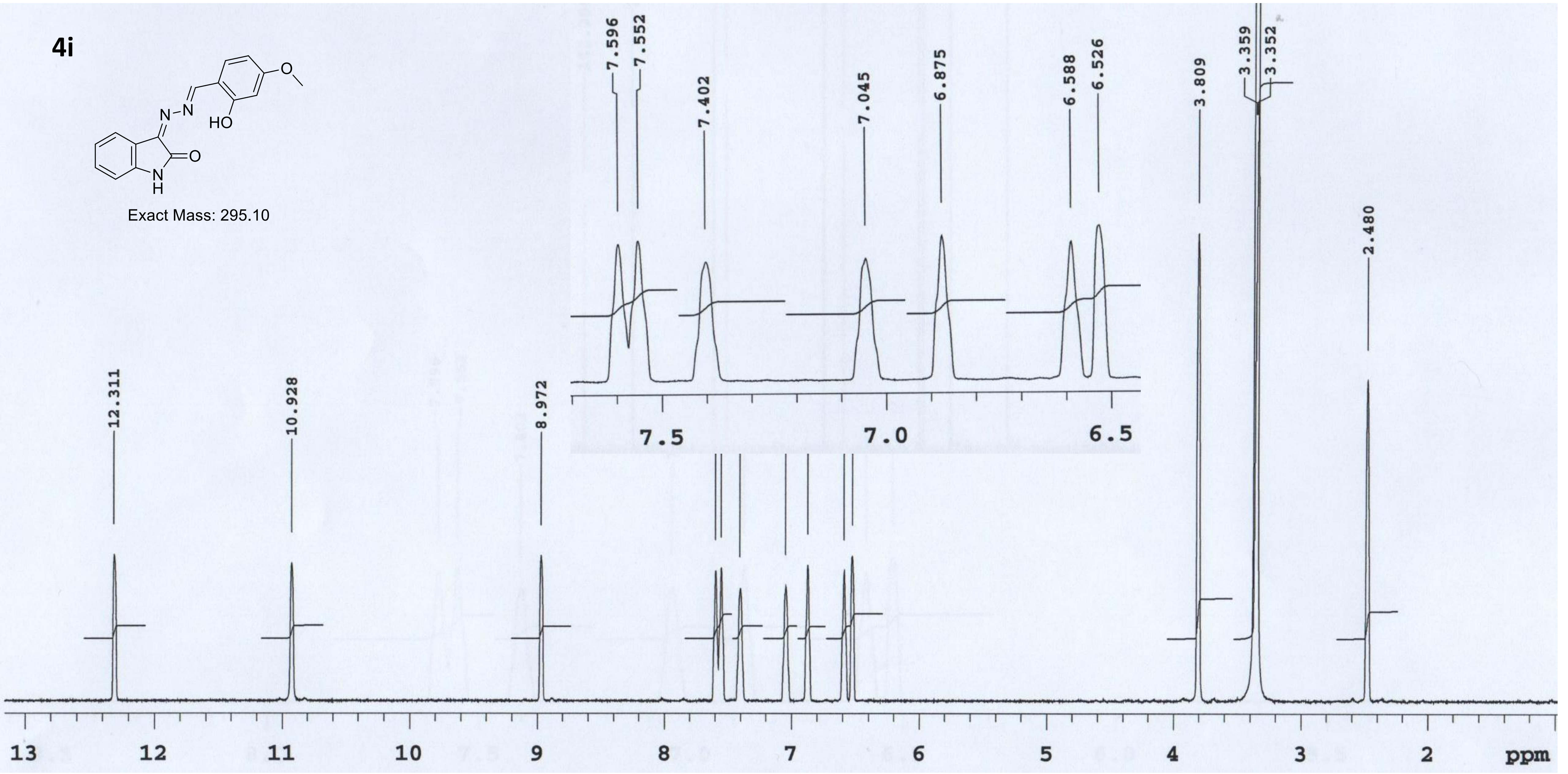

Figure S7. Proton (^{1}H) Spectra of **4i**

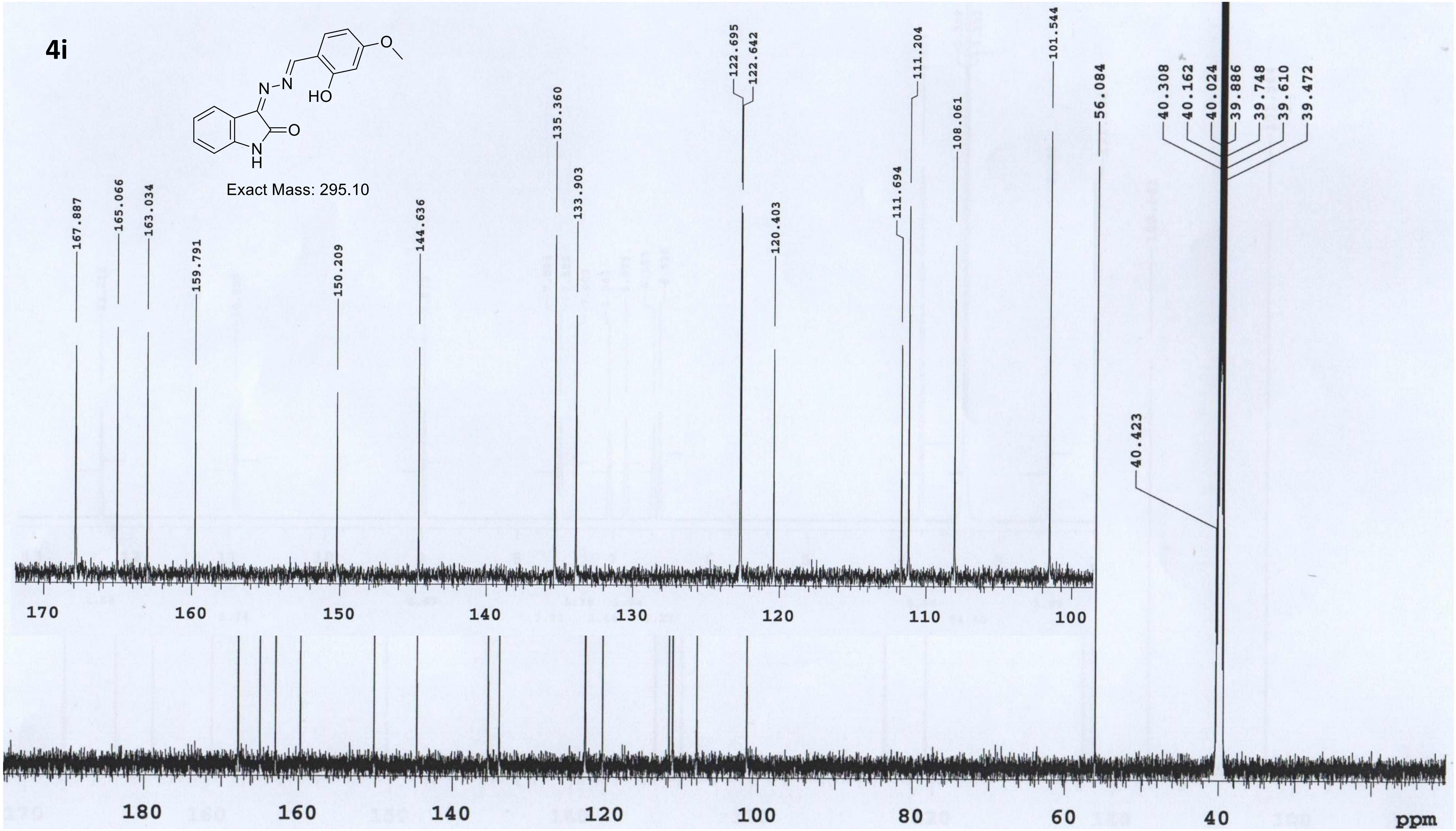

Figure S8. Carbon (^{13}C) Spectra of **4i**

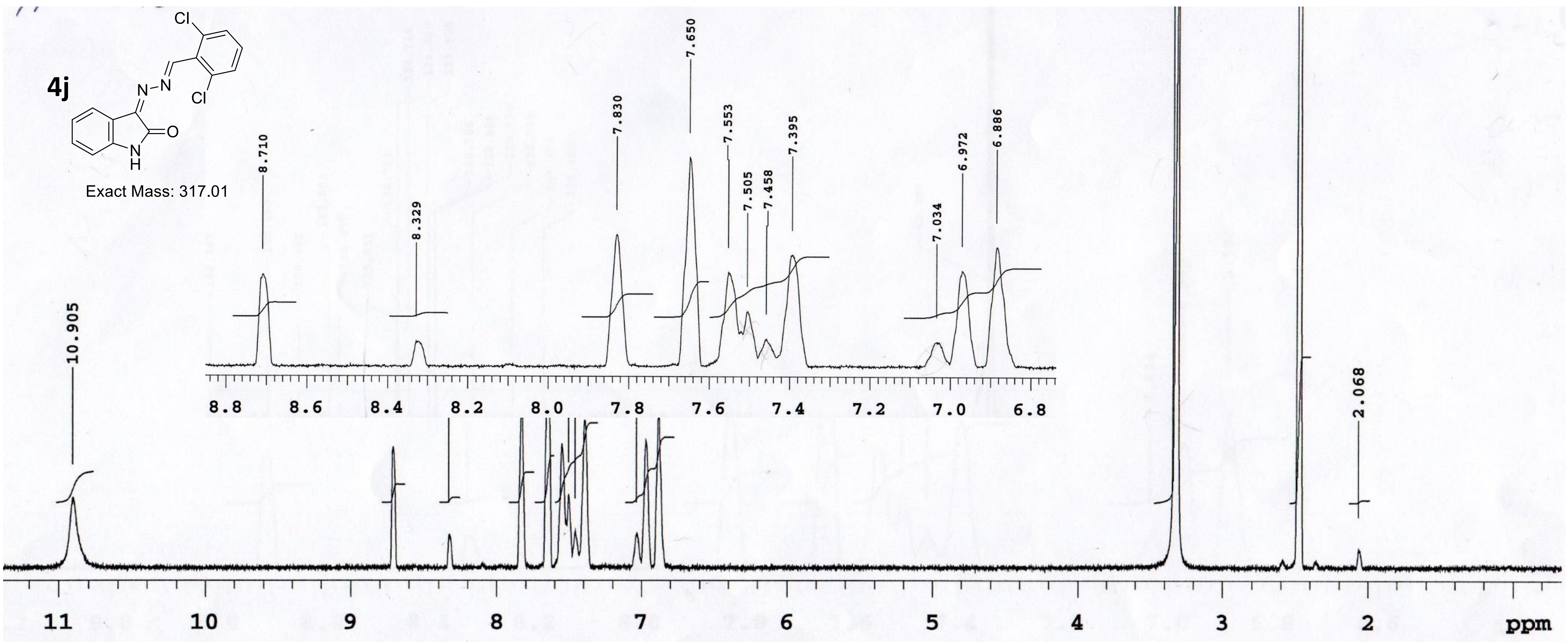

Figure S9. Proton (^{1}H) Spectra of **4j**

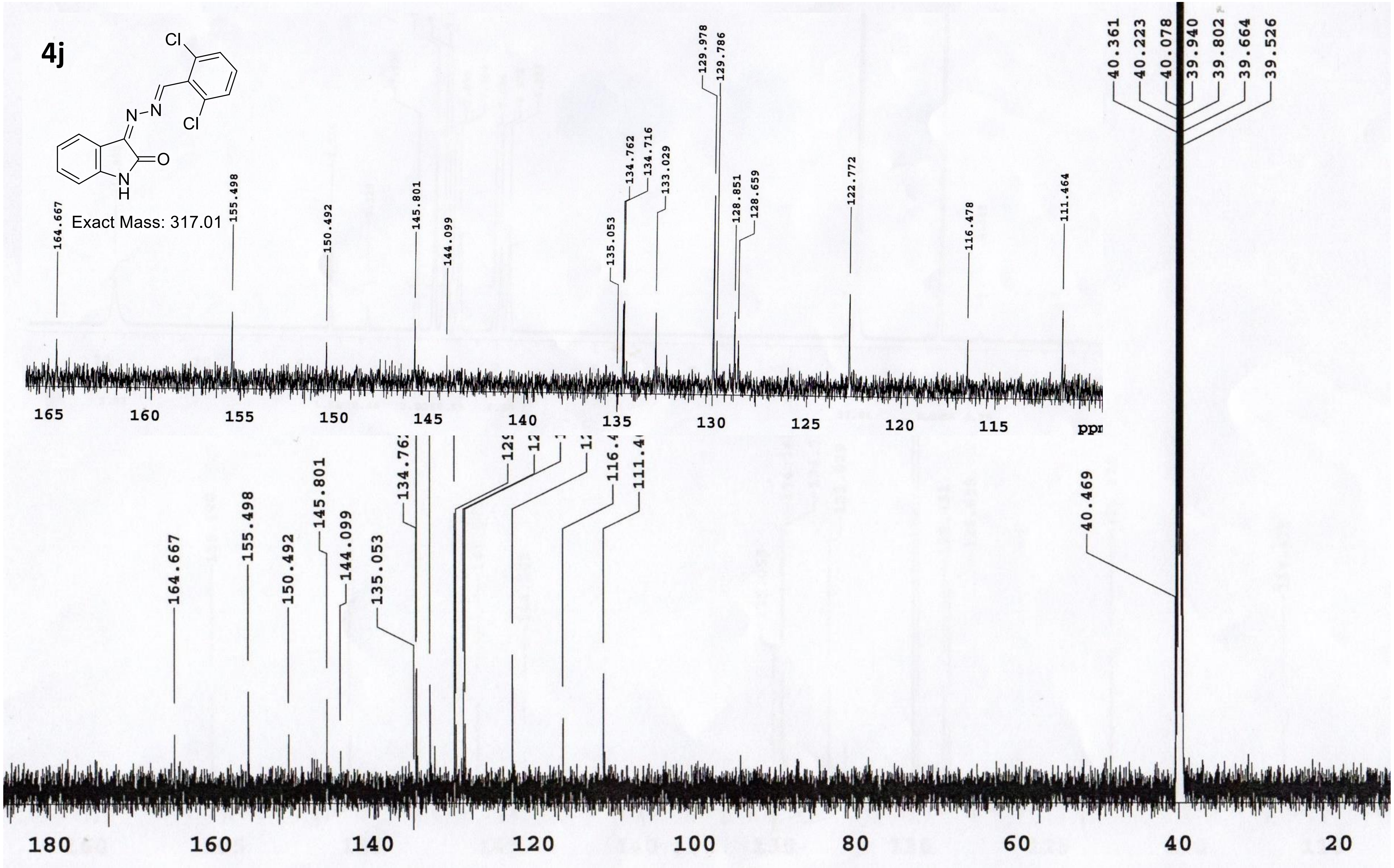

Figure S10. Carbon (^{13}C) Spectra of **4j**

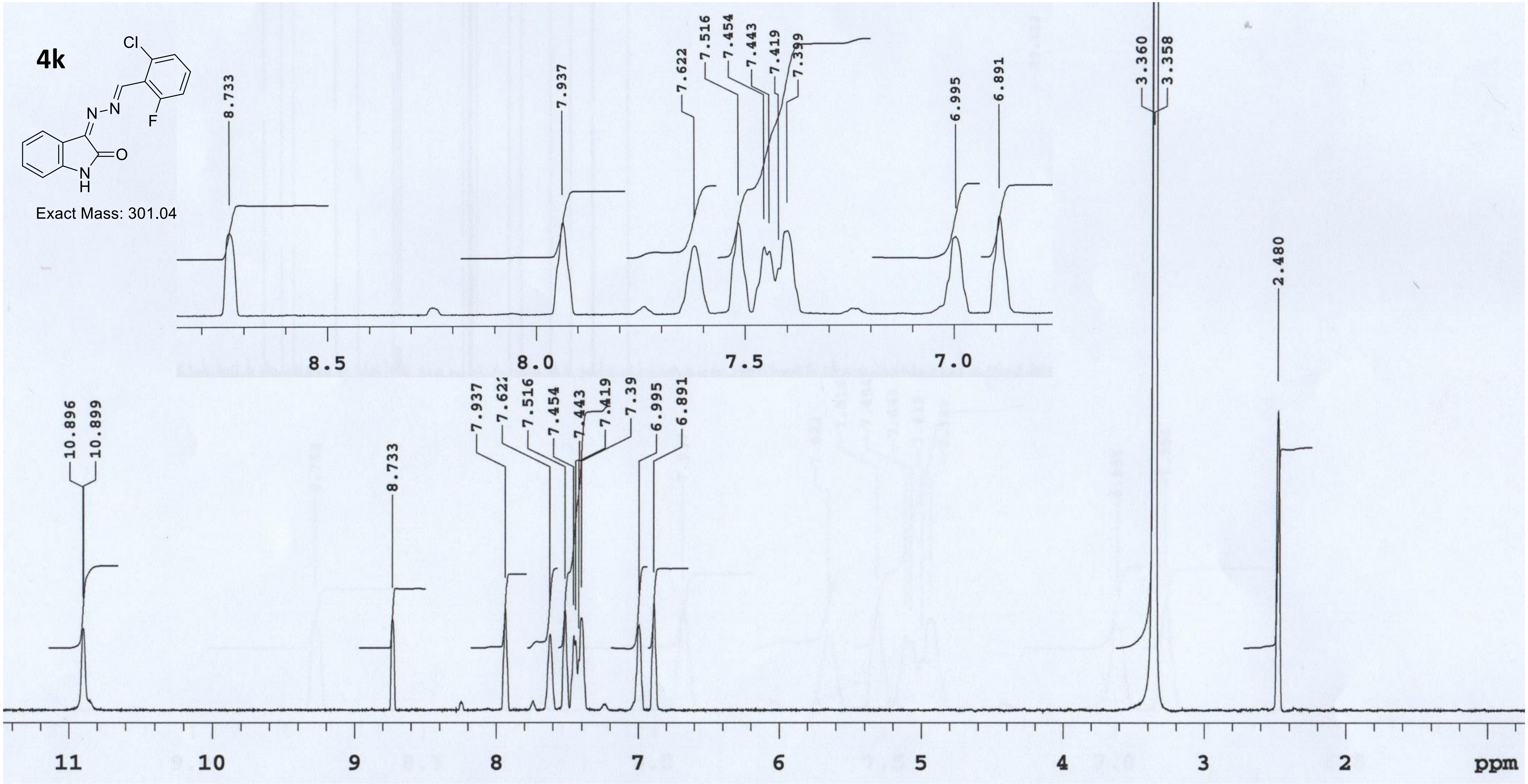

Figure S11. Proton (^{1}H) Spectra of **4k**

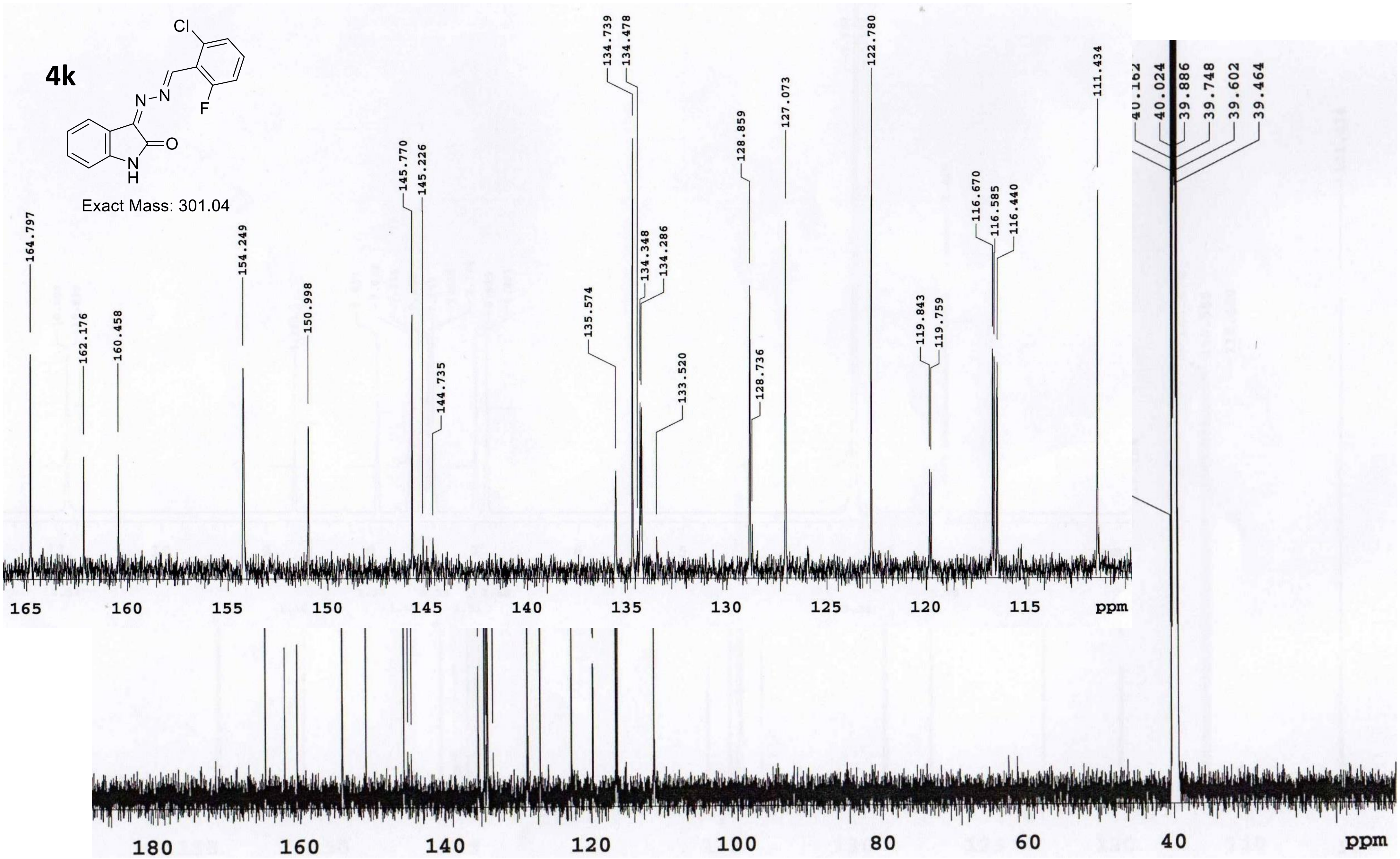

Figure S12. Carbon (^{13}C) Spectra of **4k**

4a

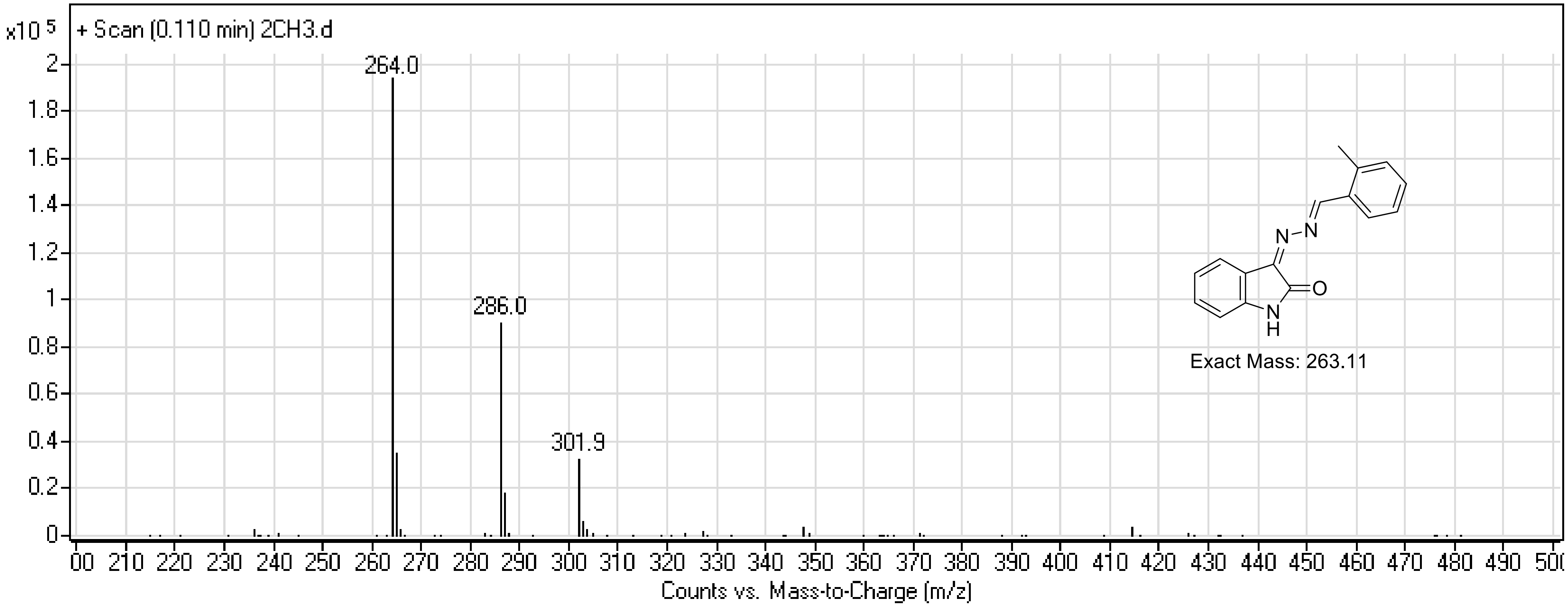

Figure S13. Mass Spectra of **4a**

4b

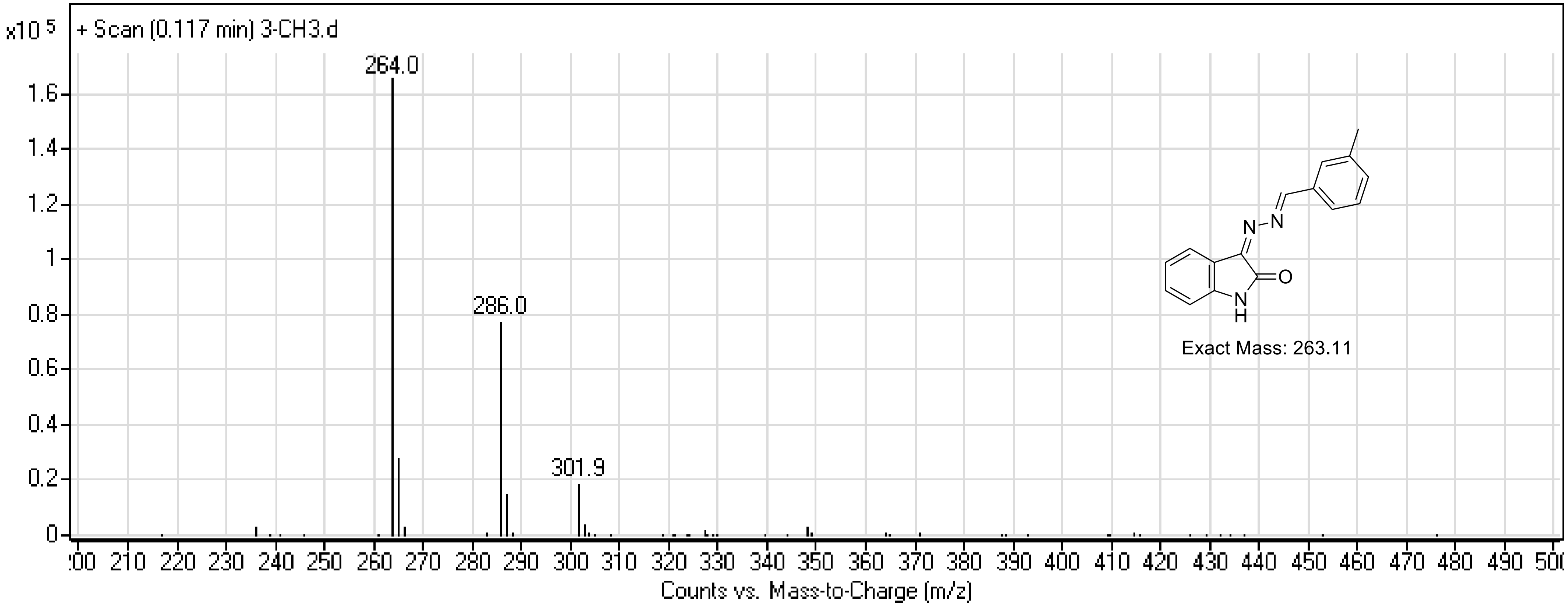

Figure S14. Mass Spectra of **4b**

4c

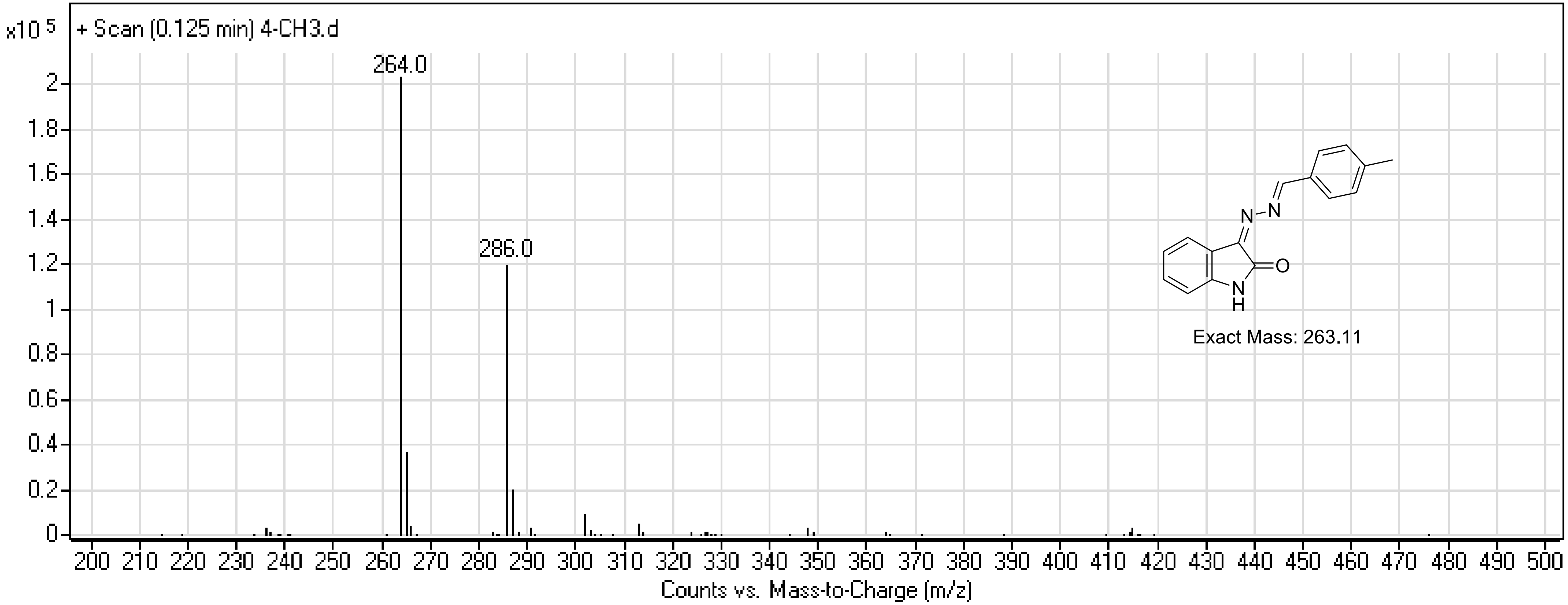

Figure S15. Mass Spectra of **4c**

4d

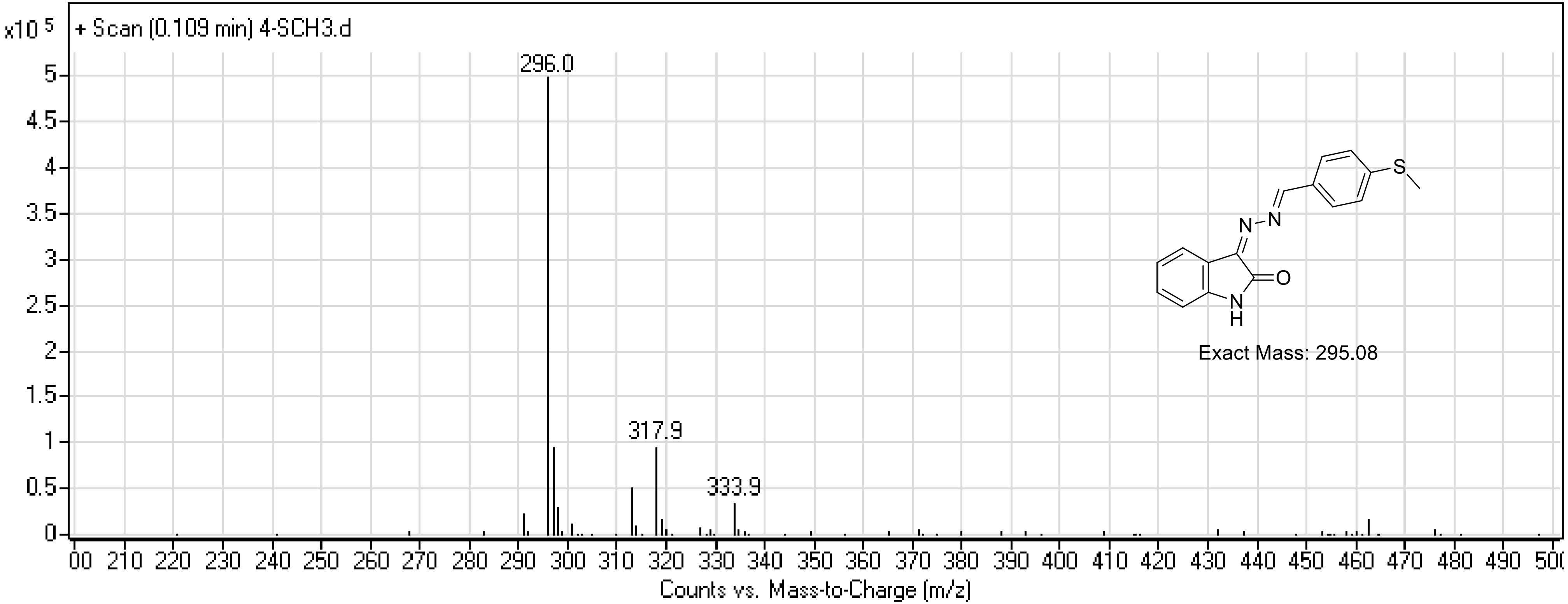

Figure S16. Mass Spectra of **4d**

4e

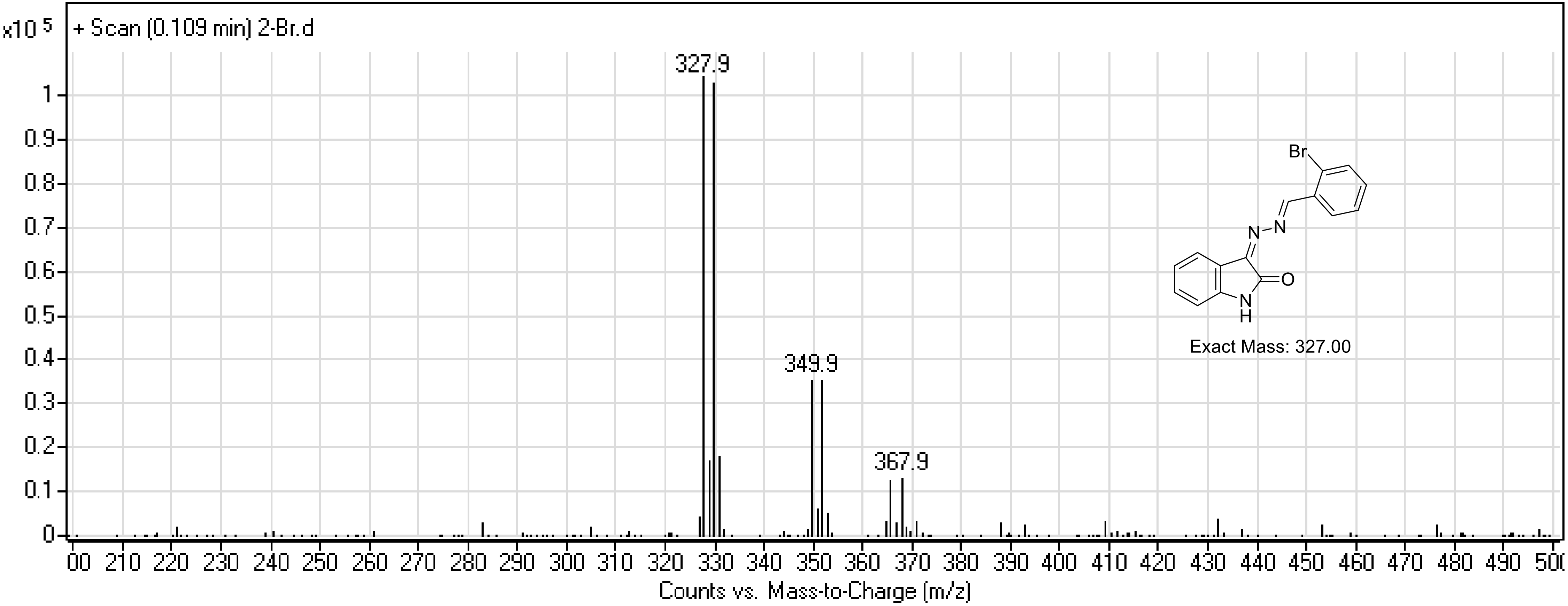

Figure S17. Mass Spectra of **4e**

4f

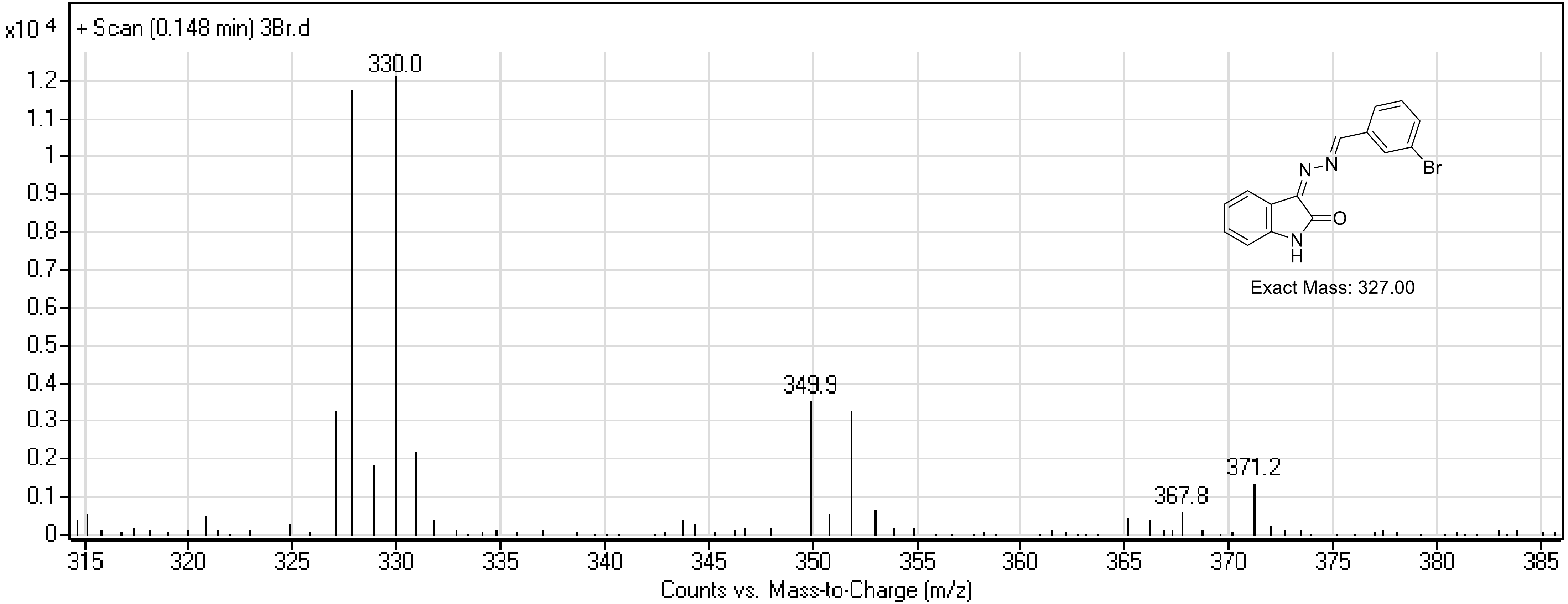

Figure S18. Mass Spectra of **4f**

4g

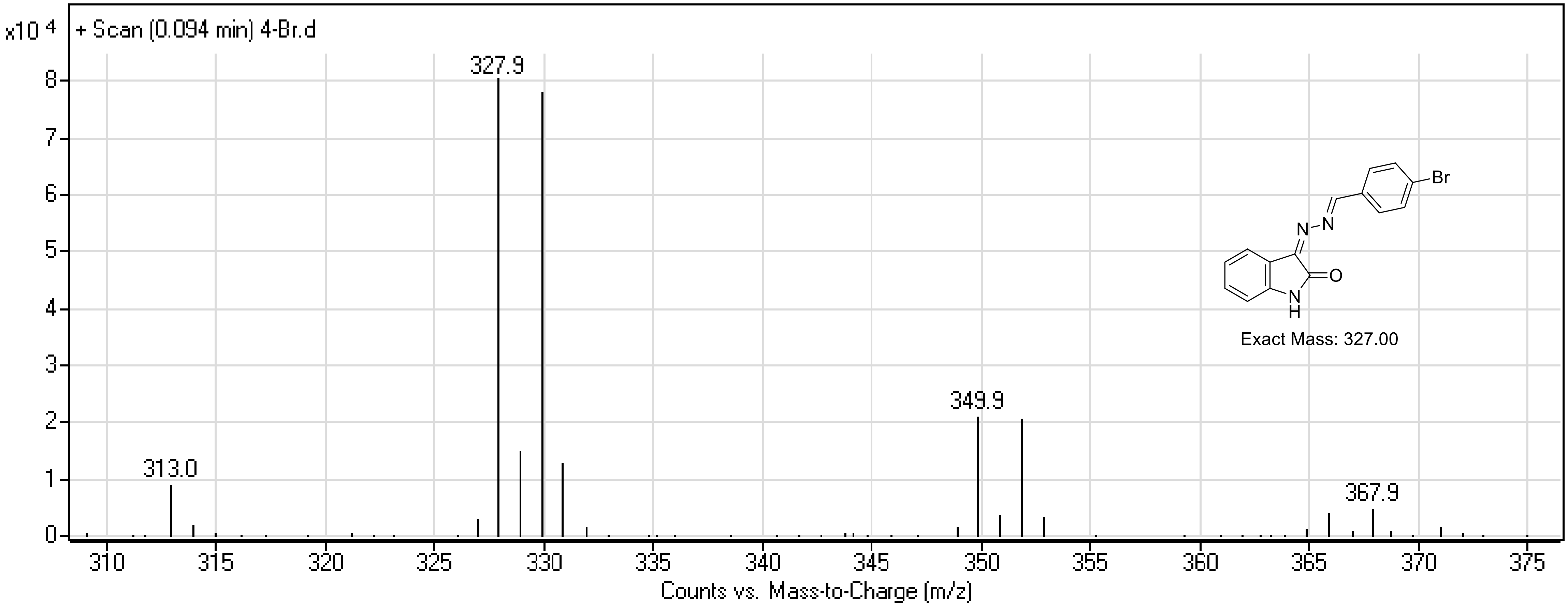

Figure S19. Mass Spectra of **4g**

4h

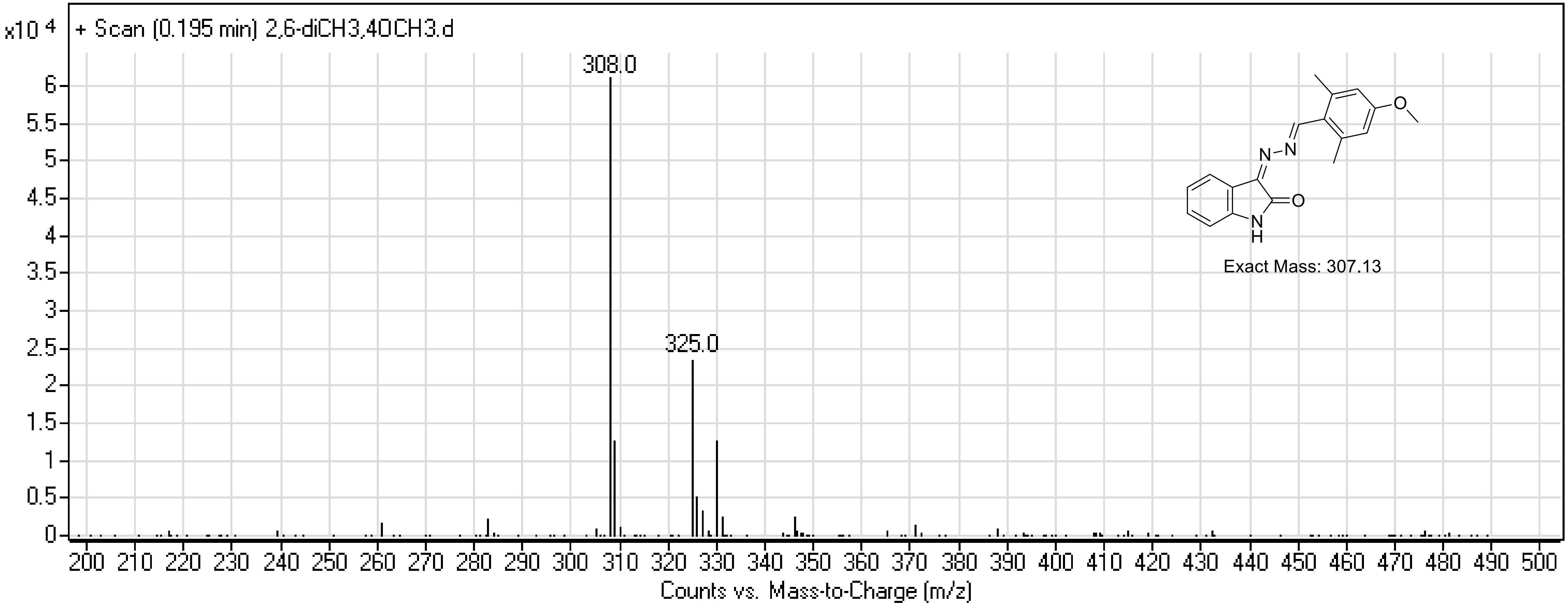

Figure S20. Mass Spectra of **4h**

4i

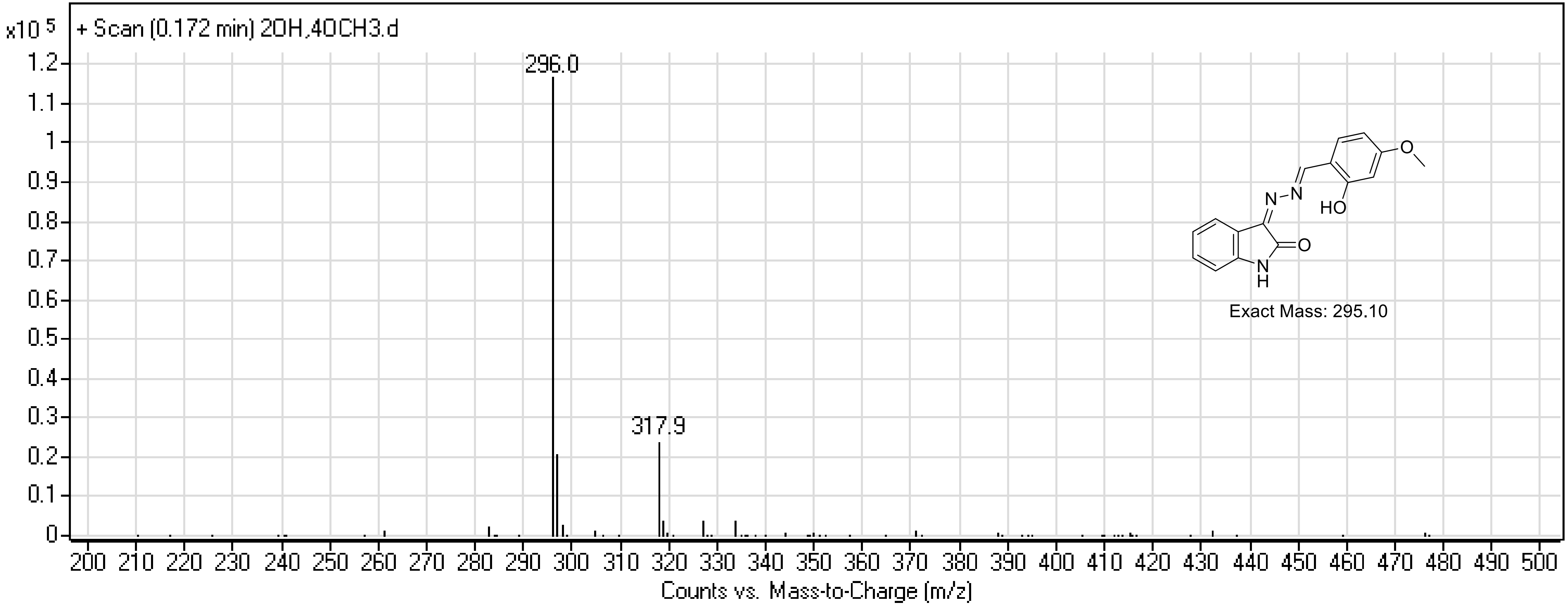

Figure S21. Mass Spectra of **4i**

4j

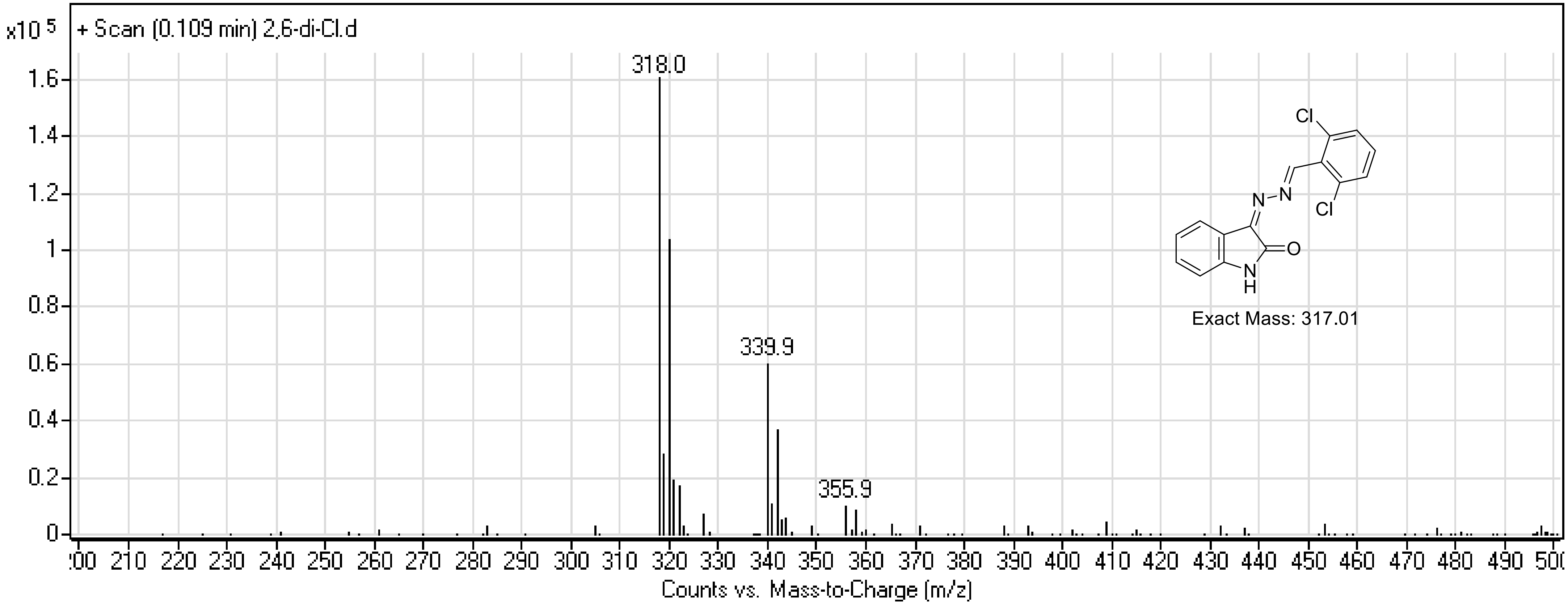

Figure S22. Mass Spectra of **4j**

4k

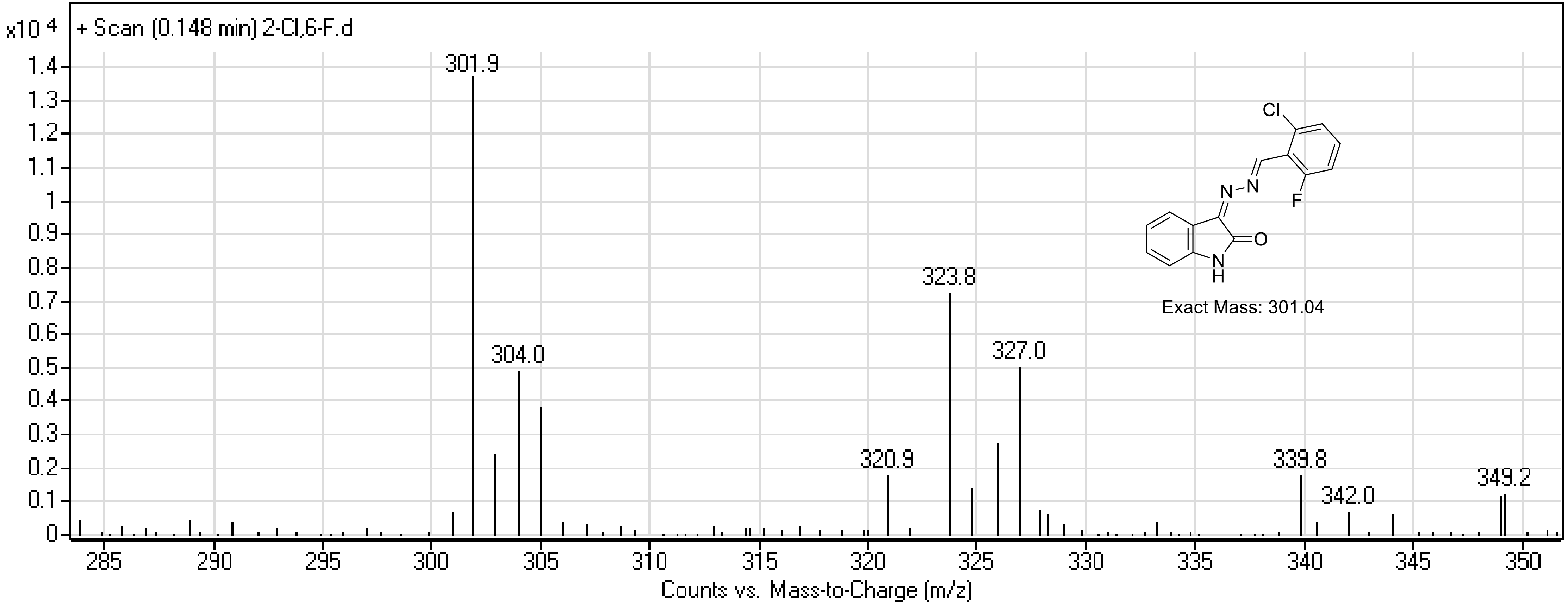

Figure S23. Mass Spectra of **4k**
